# Dual mechanism underlying failure of neural tube closure in the *Zic2* mutant mouse

**DOI:** 10.1242/dmm.049858

**Published:** 2023-03-14

**Authors:** Sarah Escuin, Saba Rose Raza-Knight, Dawn Savery, Carles Gaston-Massuet, Gabriel L. Galea, Nicholas D. E. Greene, Andrew J. Copp

**Affiliations:** Developmental Biology and Cancer Department, Great Ormond Street Institute of Child Health, University College London, London WC1N 1EH, UK

**Keywords:** Embryo, Neurulation, Neural tube defects, Actomyosin, BMP, Morphogenesis

## Abstract

Understanding the molecular mechanisms that lead to birth defects is an important step towards improved primary prevention. Mouse embryos homozygous for the *Kumba* (*Ku*) mutant allele of *Zic2* develop severe spina bifida with complete lack of dorsolateral hinge points (DLHPs) in the neuroepithelium. Bone morphogenetic protein (BMP) signalling is overactivated in *Zic2^Ku/Ku^* embryos, and the BMP inhibitor dorsomorphin partially rescues neural tube closure in cultured embryos. RhoA signalling is also overactivated, with accumulation of actomyosin in the *Zic2^Ku/Ku^* neuroepithelium, and the myosin inhibitor Blebbistatin partially normalises neural tube closure. However, dorsomorphin and Blebbistatin differ in their effects at tissue and cellular levels: DLHP formation is rescued by dorsomorphin but not Blebbistatin, whereas abnormal accumulation of actomyosin is rescued by Blebbistatin but not dorsomorphin. These findings suggest a dual mechanism of spina bifida origin in *Zic2^Ku/Ku^* embryos: faulty BMP-dependent formation of DLHPs and RhoA-dependent F-actin accumulation in the neuroepithelium. Hence, we identify a multi-pathway origin of spina bifida in a mammalian system that may provide a developmental basis for understanding the corresponding multifactorial human defects.

## Teaser

**Summary:** We identify a dual mechanism of spina bifida in *Zic2* mutant mice – a possible paradigm for multifactorial human defects. BMP overactivation prevents neuroepithelial bending, whereas RhoA overactivation causes actomyosin accumulation.

## INTRODUCTION

Neural tube closure is a fundamental event of embryonic morphogenesis in which the neural plate closes dorsally to form the neural tube, the precursor of the brain and spinal cord. Failure of neural tube closure generates ‘neural tube defects’ (NTDs) – common, severe malformations that include anencephaly and open spina bifida. The elucidation of embryonic mechanisms that lead to faulty neural tube closure is important not only for an understanding of the developmental origin of human NTDs – to predict possible predisposing genetic and non-genetic factors – but also to suggest novel approaches for primary prevention of NTDs.

Over 250 different mouse genes yield NTDs when mutated (Harris and Juriloff, 2010), and, although the majority of cases involve loss of gene function, often manifesting as a phenotype in a knockout mouse strain, NTDs also occur in gene gain-of-function and overexpression models. Together, these findings demonstrate the molecular complexity of neural tube closure in mammals. However, only a few of these NTD genetic models are understood in terms of the pathogenic processes that lead to failure of neural tube closure. Examples are genes of the planar cell polarity pathway (e.g. *Vangl2*, *Celsr1*) that regulate the vital early step of neural plate shaping (‘convergent extension’) (Galea et al., 2018, 2021; Wallingford and Harland, 2002; Ybot-Gonzalez et al., 2007b), and genes of the grainyhead-like (*Grhl2/3*) and integrin families (*Itgb1*) that determine the biomechanics of the surface ectoderm, an essential tissue component of neural tube ‘zippering’ closure (Molè et al., 2020; Nikolopoulou et al., 2019). In this paper, we explore the developmental mechanisms underlying neurulation failure in mice lacking the *Zic2* gene.

*Zic2* encodes a transcription factor that controls expression of many key developmental genes, through both DNA binding and protein–protein interactions (Diamand et al., 2018; Houtmeyers et al., 2013). Early developmental pluripotency is regulated by *Zic2*, alongside its family member *Zic3*, while the emergence of the neural cell lineage involves key interactions between *Zic2* and genes including *Sox2* and *Otx2*. In humans, *ZIC2* mutations are the second-most-common known cause (after *SHH* mutations) of the severe brain defect holoprosencephaly, in which the cerebral hemispheres fail to separate fully, owing to faulty midline specification (Barratt and Arkell, 2018). In human malignancy, *ZIC2* overexpression has been identified in gastric, endometrial and squamous cell tumours (Houtmeyers et al., 2018).

In mice, *Zic2* is expressed throughout the epiblast at gastrulation, with particular expression in the node and head process mesendoderm (Elms et al., 2004). As neurulation begins, *Zic2* becomes localised to the developing neural tube and migrating neural crest (Elms et al., 2003), with progressive dorsalisation of neural tube expression (Gaston-Massuet et al., 2005). *Zic2* loss of function leads to NTDs affecting both the cranial and spinal regions (Elms et al., 2003; Nagai et al., 2000). The ethyl-nitrosourea (ENU)-induced *Kumba* (*Zic2^Ku^*) mutant allele harbours a loss-of-function missense mutation (A1350T), which results in a cysteine-to-serine substitution at amino acid 370, disrupting the fourth zinc finger domain of Zic2 (Elms et al., 2003). Previously, we showed that *Zic2^Ku/Ku^* embryos fail to form dorsolateral hinge points (DLHPs) in the spinal neuroepithelium, exhibiting faulty closure beginning at the stage when DLHPs first appear in wild-type littermates (Ybot-Gonzalez et al., 2007a).

DLHPs are paired focal bending points in the closing neural tube at low spinal levels that involve a neuroepithelial bending angle of at least 75° (Ybot-Gonzalez et al., 2002), with localisation at ∼60% of the distance (ventromedial to dorsolateral) along the neural plate. This position marks the transition of the neuroepithelium from basal contact with mesoderm to surface ectoderm (McShane et al., 2015). DLHP formation requires that the action of BMPs secreted by the dorsal surface ectoderm is suppressed by BMP antagonists including noggin, which are produced from the dorsal neuroepithelium, under negative regulation by Shh (Ybot-Gonzalez et al., 2002; Ybot-Gonzalez et al., 2007a). We found that noggin and neuralin, another BMP antagonist, are decreased in *Zic2^Ku/Ku^* spinal neural folds, whereas phosphorylated (p)Smad-1,5,8 complex is increased in abundance (Ybot-Gonzalez et al., 2007a).

This work suggested a possible overactivation of BMP signalling in *Zic2^Ku/Ku^* embryos that might prevent DLHP formation leading to open spina bifida. However, this hypothesis has not been tested, and other possible neurulation mechanisms (e.g. cytoskeleton-related) have yet to be explored in the *Zic2^Ku^* spina bifida model. Here, we describe a dual mechanism of spinal NTDs in this mouse, in which BMP-overactivation and RhoA-dependent actomyosin accumulation defects co-exist, both contributing to the neurulation failure that characterises *Zic2^Ku/Ku^* embryos, and each requiring distinct interventions to be ameliorated.

## RESULTS

*Zic2^Ku^* mutant mice were studied initially on a congenic C57BL/6 genetic background, but homozygous *Zic2^Ku/Ku^* embryos showed relatively early onset of growth retardation, and lethality by embryonic day (E)11.5. We therefore outcrossed to the C3H/He inbred strain, and observed enhanced growth and survival of homozygotes (Fig. S1). All results in this paper are based on F2 embryos from this mixed C57BL/6×C3H/He background. *Zic2^Ku/Ku^* embryos failed to complete spinal neural tube closure in 100% of cases (17/17 at E10.5), with development of open spina bifida as described previously (Elms et al., 2003; Ybot-Gonzalez et al., 2007a). Among *Zic2^Ku/+^* heterozygotes, open spina bifida was observed in ∼10% (3/29), with the remaining mice appearing normal.

### Arrest of spinal closure in *Zic2* mutants at a stage when DLHPs normally appear

**Fig. 1.**
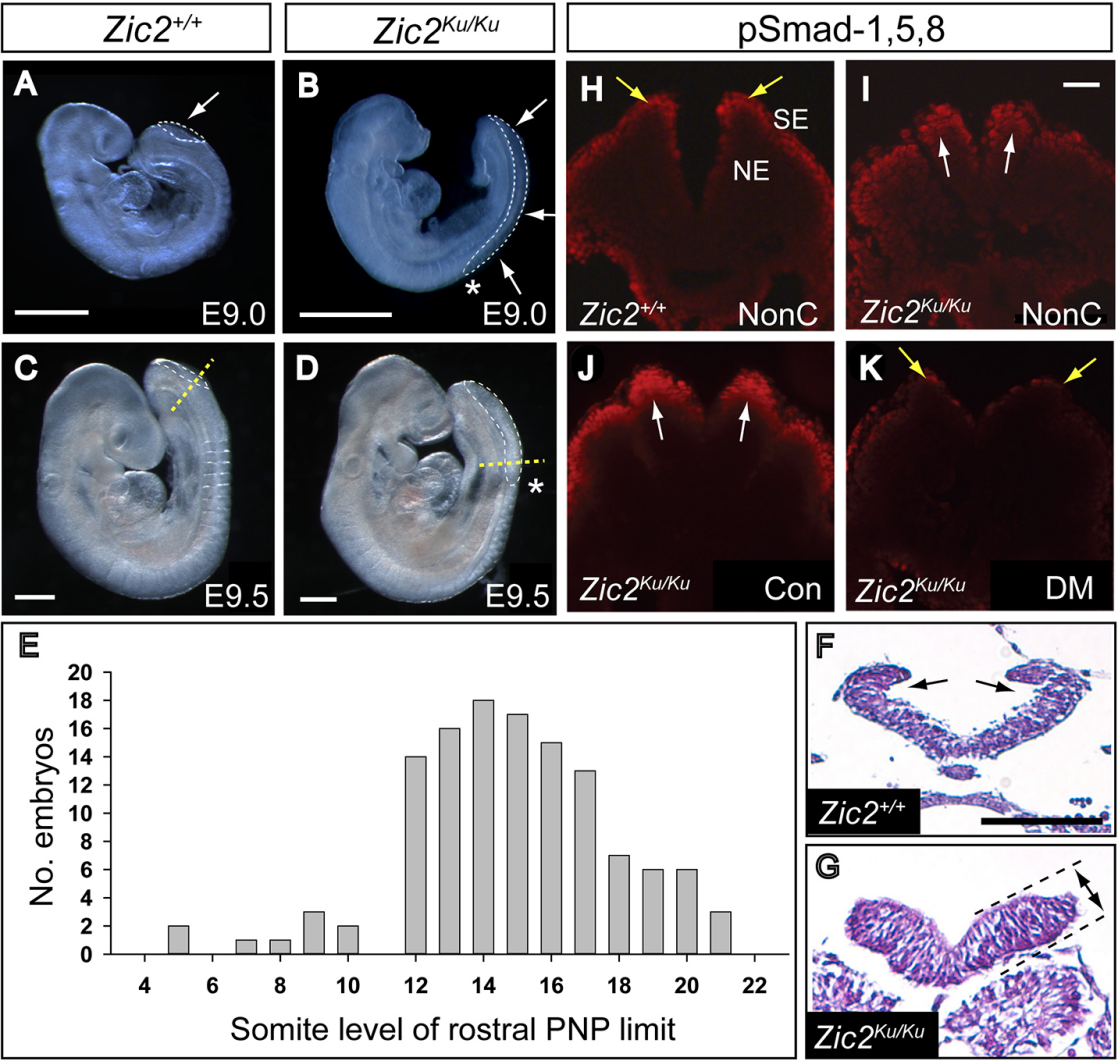
**Spinal neurulation failure and enhanced pSmad-1,5,8 expression in *Zic2^Ku/Ku^* mutants.** (A-D) Defective spinal closure, as shown by the enlarged posterior neuropore (PNP) (outlined by white dashed lines; arrows in A,B), in *Zic2^Ku/Ku^* embryos at E9.0 (B) and E9.5 (D) compared with normally developing wild-type (*Zic2^+/+^*) littermates (A,C). (E) Somite level of the rostral limit of the PNP (asterisks in B,D) among 124 *Zic2^Ku/Ku^* embryos at E9.5-10.5. Closure does not usually progress beyond the level of somites 12-17, at which level dorsolateral hinge points (DLHPs) first appear in normal development. (F,G) Transverse sections (Haematoxylin and Eosin-stained) through the rostral PNP of E9.5 *Zic2^+/+^* (F) and *Zic2^Ku/Ku^* (G) embryos, at the level of the yellow dashed lines in C,D, respectively. Both show equivalent neural fold elevation, but paired DLHPs are present in the *Zic2^+/+^* embryo (arrows in F) and absent from the *Zic2^Ku/Ku^* embryo (G). The mutant neuroepithelium shows marked apicobasal thickening (double-headed arrow between dashed lines in G). (H-K) Immunohistochemistry for pSmad-1,5,8 on transverse sections through the elevated neural folds of *Zic2^+/+^* (H) and *Zic2^Ku/Ku^* (I-K) embryos. In non-cultured (NonC) 15-somite-stage embryos (H,I), immunoreactivity is enhanced in the dorsal *Zic2^Ku/Ku^* neuroepithelium (arrows in I), compared with that in *Zic2^+/+^* embryos, in which expression is mainly in surface ectoderm (arrows in H). *Zic2^Ku/Ku^* embryos exposed for 16 h in culture to vehicle (Con) from E8.5 show pSmad-1,5,8 immunoreactivity comparable to that seen in non-cultured *Zic2^Ku/Ku^* embryos (compare J with I). Dorsomorphin exposure in culture produces markedly reduced pSmad-1,5,8 expression (K), similar to that in non-cultured *Zic2^+/+^* embryos. NE, neuroepithelium; SE, surface ectoderm. Scale bars: 0.5 mm (A-D); 50 µm (F); 50 µm (I).

Analysis at E9.5 revealed severely abnormal progression of posterior neuropore (PNP) closure in the spinal region of *Zic2^Ku/Ku^* embryos. The PNP first appears at the six-somite stage during normal development, following initiation of closure at the hindbrain/cervical boundary (i.e. Closure 1). Closure then progresses by ‘zippering’ along the spinal region with gradual reduction in PNP length (van Straaten et al., 1992). Primary neurulation ends at around the 30-somite stage, when PNP closure is completed in the future sacral region (Copp and Brook, 1989; Copp et al., 1982). In contrast to this normal sequence, *Zic2^Ku/Ku^* embryos exhibit arrest in the progression of spinal closure as indicated by an enlarged PNP (Fig. 1A-D) and progressive increase in PNP length with somite number (Fig. S2). The majority of embryos (75%) show a rostral PNP limit (i.e. where spinal closure arrests) at the level of somites 12-17 (Fig. 1E), in contrast to non-mutant E9.5 embryos in which the rostral PNP limit is always caudal to the somite rows, i.e. flanked by pre-somitic mesoderm. The axial position of PNP closure arrest in mutants corresponds to the transition between Modes 1 and 2 of spinal neurulation, when neural plate bending at DLHPs first appears in normal embryos (Shum and Copp, 1996). *Zic2^Ku/Ku^* embryos completely lack DHLPs, in contrast to wild-type littermates (Fig. 1F,G), whereas a median hinge point (MHP) is present in both. Another consistent feature of the *Zic2^Ku/Ku^* neuroepithelium is apicobasal thickening (Fig. 1F,G), which may be causally related to the lack of DLHPs. Hence, mouse embryos lacking Zic2 function specifically fail in DLHP development, and this seems to be a likely cause of spinal closure arrest and origin of spina bifida in these embryos.

### BMP/Smad signalling is overactivated during *Zic2^Ku^* mutant spinal neurulation

*Zic2* is expressed throughout the neuroepithelium, before PNP closure (Fig. S3A,B), remains expressed in almost all of the neural tube soon after closure (Fig. S3C), and then shows restriction of expression to the dorsal neural tube and rostral part of the pre-somitic mesoderm (Fig. S3D). To investigate the developmental mechanisms underlying lack of DLHPs and failure of spinal closure in *Zic2^Ku/Ku^* mutants, we re-explored an apparent overactivation of BMP/Smad signalling (Ybot-Gonzalez et al., 2007a). In this pathway, extracellular BMP binds to cell membrane receptors, which then phosphorylate Smad proteins that go to the nucleus to regulate gene expression (Wang et al., 2014). Using freshly collected embryonic samples, we confirmed that immunostaining for pSmad-1,5,8 was enhanced in the dorsal neural plate of *Zic2^Ku/Ku^* mutants, compared with wild-type littermates (Fig. 1H,I; Fig. S4A-D,M). In contrast, pSmad-1,5,8 expression did not differ between wild-type embryos and littermates homozygous for the *splotch* (*Pax3^Sp2H^*) mutation that also causes severe spina bifida (Fig. S4E-H). Hence, the presence of failing PNP closure is not responsible for elevated pSmad-1,5,8 expression, supporting the view that enhanced BMP signalling is likely to be involved specifically in the pathogenesis of neurulation defects in *Zic2^Ku/Ku^* mutants.

### Suppression of BMP signalling partially normalises closure in *Zic2^Ku^* mutants

**Fig. 2.**
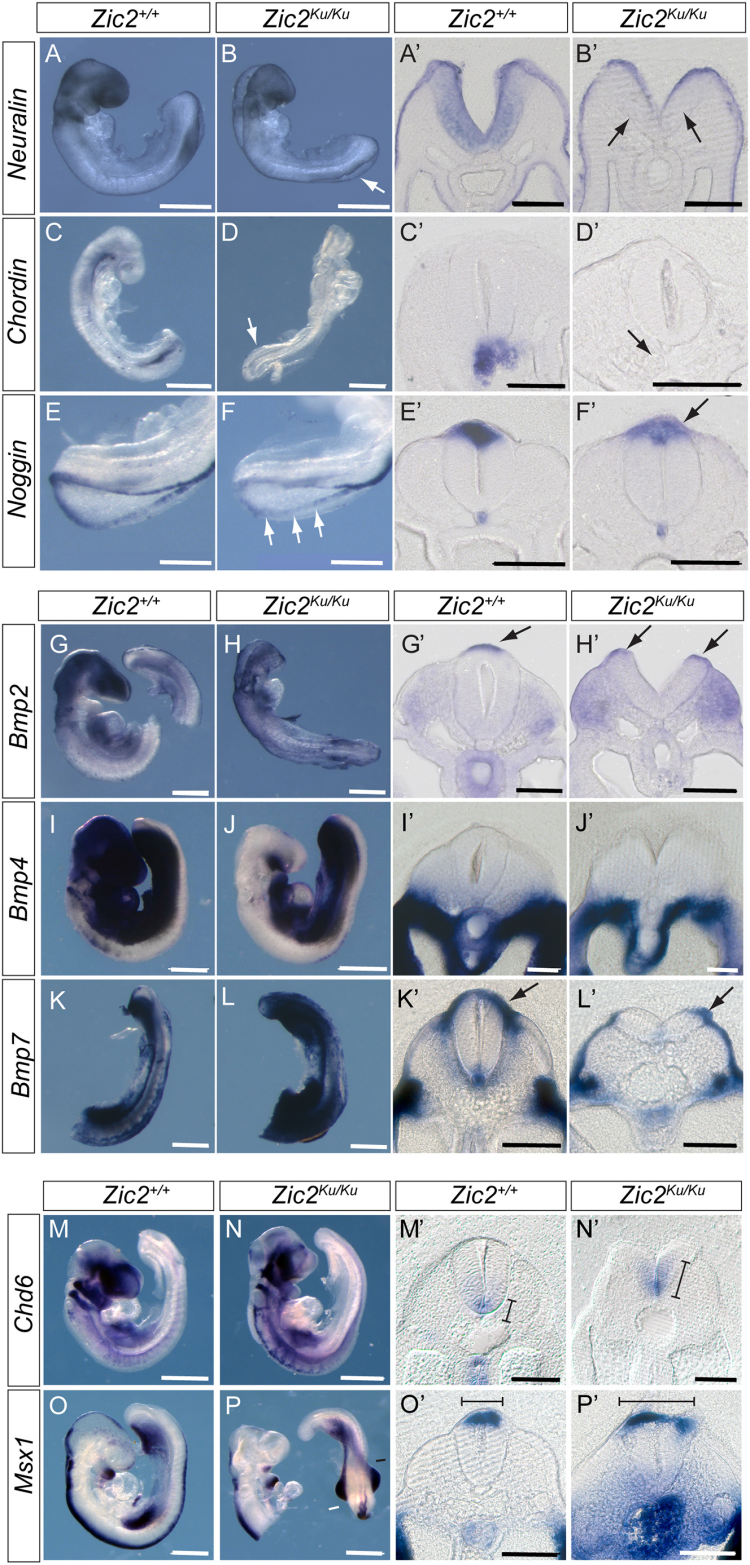
**Overactivation of BMP signalling in pre-spina bifida *Zic2^Ku/Ku^* embryos.** (A-P′) Comparison of *Zic2^+/+^* (A,A′,C,C′,E,E′,G,G′,I,I′,K,K′,M,M′,O,O′) and *Zic2^Ku/Ku^* (B,B′,D,D′,F,F′,H,H′,J,J′,L,L′,N,N′,P,P′) embryos at E9.5 for expression of BMP antagonists (A-F′), BMP ligands (G-L′) and downstream markers of BMP signalling (M-P′). Whole-mount *in situ* hybridisation was followed by vibratome sectioning through the closing or recently closed spinal neural tube. (A-F′) BMP antagonists neuralin, chordin and noggin are expressed on the tips of the neural folds throughout the PNP, and all show reduced expression in *Zic2^Ku/Ku^* embryos compared with wild-type littermates (arrows in B,B′,D,D′,F,F′). (G-L′) In contrast, *Bmp2* and *7* show no marked differences in dorsal expression intensity between genotypes (arrows in G′,H′,K′,L′), while *Bmp4* is not expressed dorsally (I′,J′). Apparent increase in cranial expression of *Bmp4* in the wild-type embryo (I) is due to probe trapping. (M-P′) Domains of *Chd6* and *Msx1* expression in the neural tube, which are regulated by BMP signalling, are extended in *Zic2^Ku/Ku^* embryos compared with wild-type embryos (lines in M′,N′,O′,P′). Strong signal for *Msx1* in the gut of the mutant embryo (P′) is due to probe trapping. Three embryos were hybridised for each probe and genotype combination except for *Bmp2* where *n*=2, with representative images shown. Scale bars: 0.5 mm (A-D,G-P); 1 mm (E,F); 50 µm (A′-L′); 100 µm (M′-P′).

**Fig. 3.**
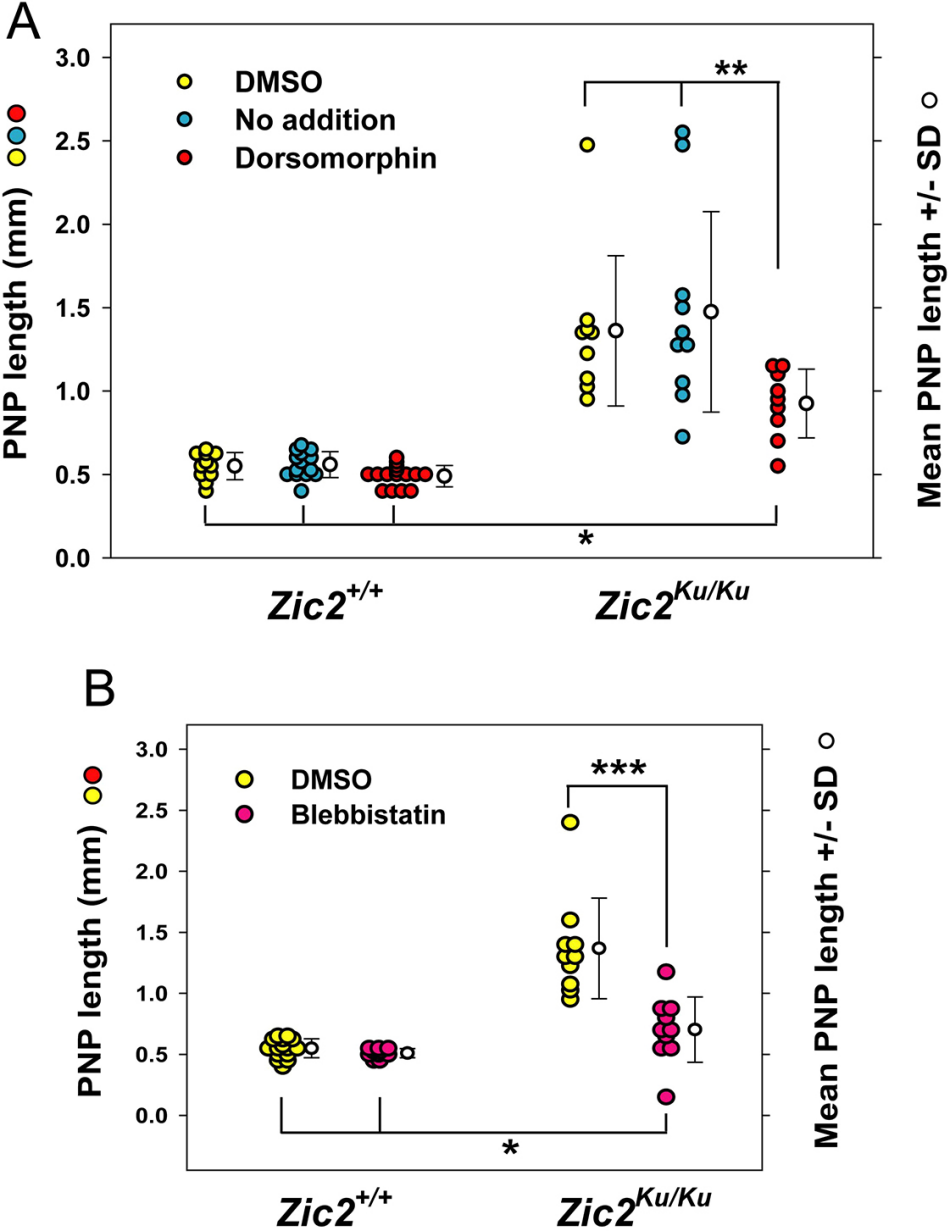
**Partial rescue of spinal NTDs in *Zic2^Ku/Ku^* embryos by inhibitors of BMP signalling and actomyosin accumulation.** (A,B) Embryos were cultured for 18 h from E8.5 to the 15- to 19-somite stage, with addition of dorsomorphin (DM) to inhibit BMP signalling (A), or Blebbistatin (Bleb) to inhibit myosin II (B). *Zic2^+/+^* embryos exhibit normal spinal neural tube closure, with PNP lengths of ∼0.5 mm, which is typical of this stage, whether untreated (‘no addition’ in A), cultured with DMSO vehicle, or after administration of either DM or Bleb. In contrast, untreated and DMSO-treated *Zic2^Ku/Ku^* embryos show significantly enlarged PNPs (*P*<0.001), with lengths of 1.0-2.5 mm, an indication of incipient spina bifida. Both DM (A) and Bleb (B) cause a significant reduction in PNP length in *Zic2^Ku/Ku^* embryos compared with DMSO-treated controls (**P<0.05 for DM; ***P<0.001 for Bleb). However, DM- and Bleb-treated mutant embryos have PNPs that remain significantly longer than those of wild-type embryos, whether untreated, DMSO- or inhibitor-treated (*P<0.001 for DM; *P<0.05 for Bleb). Hence, rescue of spinal neurulation in *Zic2^Ku/Ku^* embryos by DM and Bleb is incomplete. Each point on the graphs represents an individual embryo. Two-way ANOVA (genotypes; treatments) with Holm-Sidak or Dunn's post-hoc tests for pairwise differences.

**Fig. 4.**
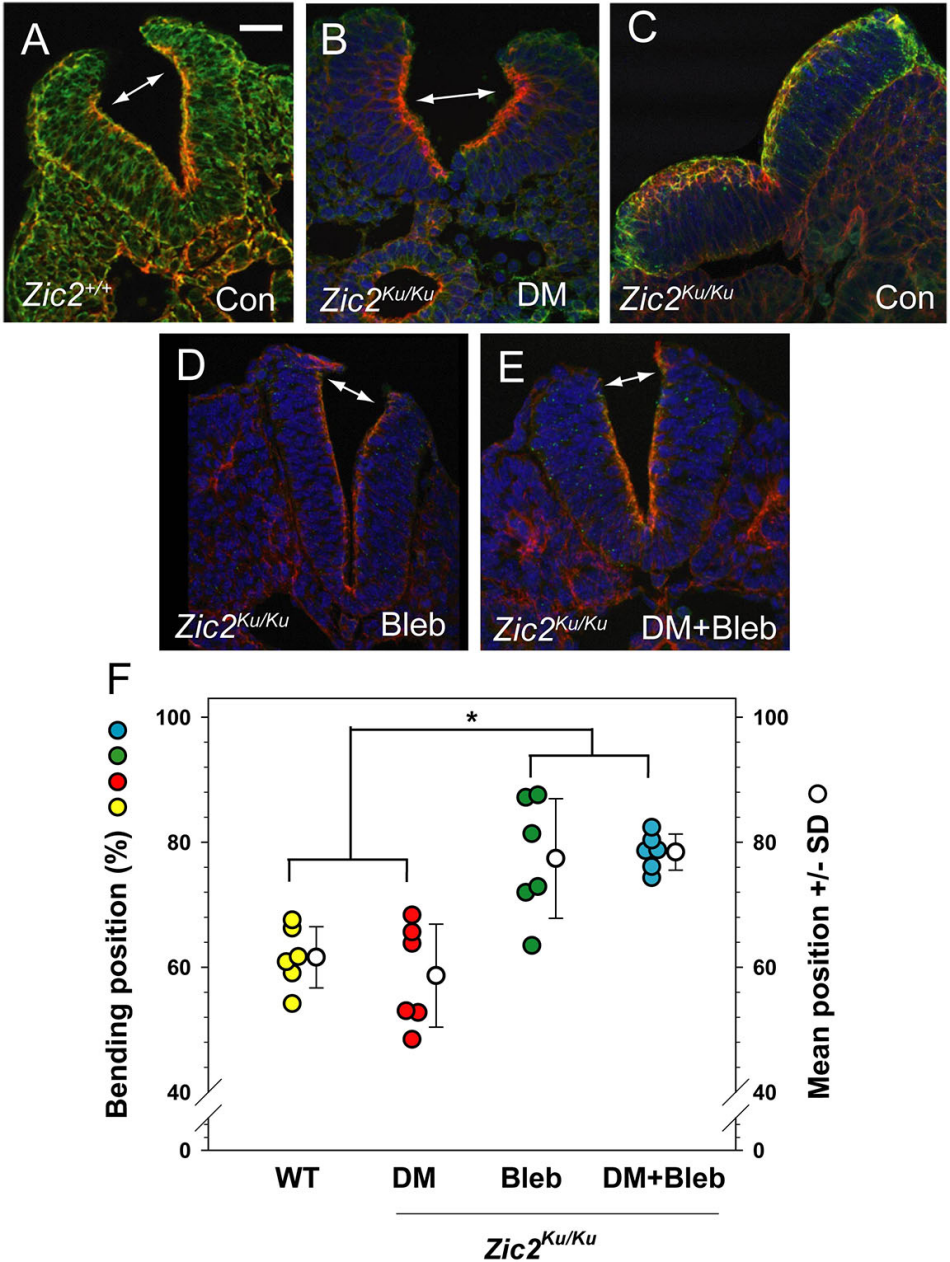
**Neural plate bending at distinct ventrodorsal positions after partial rescue of closure by DM or Bleb.** (A-E) *Zic2^+/+^*(A) and *Zic2^Ku/Ku^* (B-E) embryos at the 15- to 19-somite stage, either uncultured (A,C; Con) or cultured in the presence of DM, Bleb or both (B,D,E). Transverse sections at rostral PNP level are stained with Phalloidin (red), anti-MHC (green) and DAPI (blue). Note the typical DLHPs in the wild-type neural plate (double arrow in A), and closely similar bending points in the DM-treated mutant neural plate (double arrow in B). An untreated mutant (C) shows a complete lack of DLHPs and an apicobasally thickened neural plate. *Zic2^Ku/Ku^* embryos treated with Bleb or Bleb+DM show more distally located bending, just below the dorsal neural plate tips (double arrows in D,E). (F) Measurement of bending position, as % of ventral–dorsal (V-D) distance along the apical neural plate border. Wild-type and DM-treated mutant embryos exhibit bends clustering ∼60% of the V-D distance whereas Bleb (+/− DM) induces bends clustering ∼80% of the V-D distance (*P=0.002; one-way ANOVA with post-hoc Holm-Sidak tests). Three embryos (two neural folds per embryo) measured in each group. Scale bar: 30 µm.

*In situ* hybridisation analysis confirmed that the BMP antagonists noggin, neuralin and chordin are all downregulated in *Zic2^Ku/Ku^* embryos compared with wild-type embryos (Fig. 2A-F) (Ybot-Gonzalez et al., 2007a). In contrast, *Bmp2*, *Bmp4* and *Bmp7* did not show altered expression in the mutants (Fig. 2G-L), whereas the downstream markers of BMP signalling, *Cdh6* and *Msx1*, showed extended domains of expression in *Zic2^Ku/Ku^* embryos compared with wild-type embryos (Fig. 2M-P). These findings are consistent with increased BMP signalling in *Zic2^Ku/Ku^* mutants as a result of pathway activation downstream of BMP receptors. To test whether this enhanced BMP signalling contributes to impaired spinal closure, we exposed embryos in culture to the BMP signalling inhibitor dorsomorphin (Yu et al., 2008). pSmad-1,5,8 expression was markedly reduced in the neural plate of dorsomorphin-treated embryos (Fig. 1J,K; Fig. S4I-L). Moreover, mean PNP length was significantly diminished in *Zic2^Ku/Ku^* embryos exposed to dorsomorphin compared with dimethyl sulfoxide (DMSO)-treated controls, although PNP length was not restored to wild-type values (Fig. 3A). Strikingly, we observed apparently normal formation of DLHPs in dorsomorphin-treated *Zic2^Ku/Ku^* embryos (Fig. 4A,B). These findings suggest that overactivation of BMP/Smad signalling is the predominant cause of failure of DLHP formation in *Zic2^Ku/Ku^* embryos. Although this contributes to the delayed spinal neural tube closure, it does not account fully for the defective neurulation phenotype.

### Reduction of Shh signalling does not rescue spinal closure in *Zic2^Ku^* embryos

We asked whether signalling via the Sonic hedgehog (Shh) pathway might also be implicated in the spinal neurulation defects of *Zic2^Ku/Ku^* embryos. Shh is a negative regulator of DLHP formation (Ybot-Gonzalez et al., 2002), and so overactivation of Shh signalling could account for the lack of DLHPs in the mutants. However, we confirmed previous findings (Elms et al., 2003; Warr et al., 2008) that expression of *Shh* and its receptor *Ptc* (also known as *Ptch1*) are normal in *Zic2^Ku/Ku^* embryos (Fig. S5A-D), whereas *Gli1* shows marked downregulation (Fig. S5E,F), and *Gli2* and *Gli3* show minor reduction in expression (Fig. S5G-J). Dorsoventral patterning of the *Zic2^Ku/Ku^* spinal neuroepithelium appeared normal, as judged by immunohistochemistry for Pax3, Isl1 and Nkx6.1, even in regions of failed neural tube closure (Fig. S6), further indicating that Shh signalling is unaffected.

Despite these findings, a functional effect of Shh signalling in suppressing DLHP formation in *Zic2^Ku/Ku^* embryos remained a possibility, which we tested by breeding mice doubly mutant for *Shh* and *Zic2^Ku^*. Similar double mutants were produced in a previous analysis of forebrain development, although spinal closure was not studied (Warr et al., 2008). All nine expected genotypes occurred at approximately Mendelian frequencies, and, in E10.5 litters, all embryos of *Zic2^Ku/Ku^* genotype had open spina bifida, with lack of DLHPs, irrespective of whether they were wild-type, heterozygous or homozygous for the Shh null mutation (Fig. S7). Although only small numbers of embryos were obtained with the less frequent *Zic2/Shh* genotypes, the data are consistent with a lack of spina bifida rescue by Shh loss of function (Table S1). We conclude that the lack of DLHP formation in *Zic2^Ku/Ku^* mutants does not result from excessive Shh signalling.

### Actomyosin accumulates apically in the *Zic2* mutant neuroepithelium

We found previously that E9.5 *Zic2^Ku/Ku^* embryos show greater neural fold recoil than wild-type littermates, after laser ablation of the PNP zippering point (Galea et al., 2017). Hence, the *Zic2* mutant neuroepithelium appears to have altered biomechanics that may contribute to the arrest of spinal closure. We hypothesised that this biomechanical abnormality may be independent of the absence of DLHPs and is more likely to be related to the cytoskeletal status of the neuroepithelium. Indeed, previously, we found that inhibition of actin turnover, leading to neuroepithelial actomyosin accumulation, causes spinal NTDs in wild-type and *Cfl1* mutant mouse embryos, without loss of DLHPs (Escuin et al., 2015). We therefore asked whether apical actomyosin accumulation might also be a feature of spinal neurulation in the *Zic2^Ku^* mutant.

**Fig. 5.**
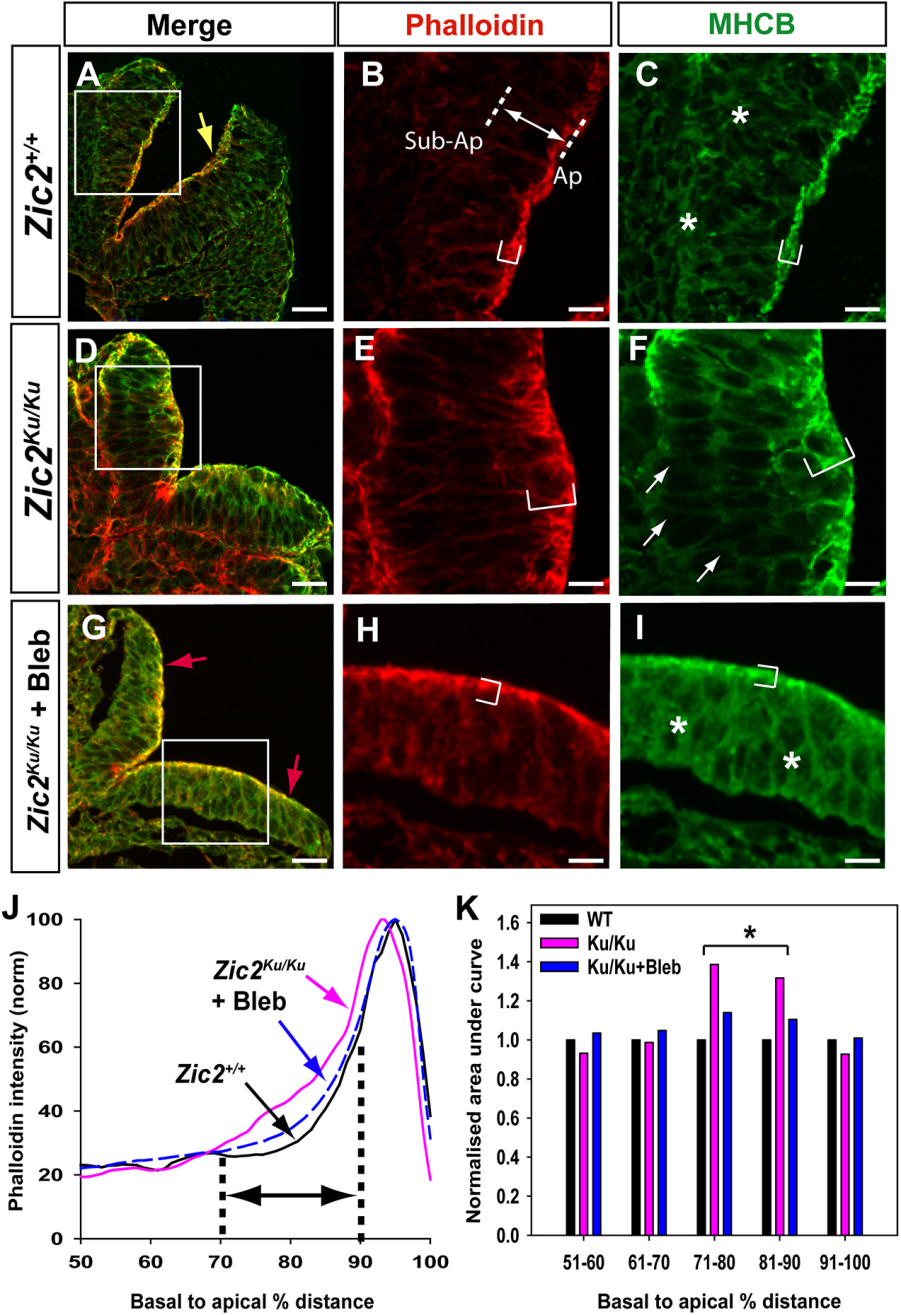
**Apical accumulation of actomyosin in pre-spina bifida *Zic2^Ku/Ku^* mutants and rescue by Bleb.** (A-I) Phalloidin staining of F-actin (red), and anti-MHCB immunostaining (green) on transverse sections through the open PNP at the 15- to 19-somite stage. Embryos are *Zic2^+/+^* (A-C) and *Zic2^Ku/Ku^* (D-F) uncultured, and *Zic2^Ku/Ku^* cultured in 50 µM Bleb for 6-8 h from the 12- to 16-somite stage (G-I). Merged images are shown at low magnification (A,D,G) with higher magnification of the boxed areas (B,C,E,F,H,I). F-actin and MHCB both occupy extended apical domains in *Zic2^Ku/Ku^* neuroepithelium compared with *Zic2^+/+^* neuroepithelium (compare bracketed regions in B,C with those in E,F). MHCB expression in sub-apical and basal neuroepithelium is diminished in *Zic2^Ku/Ku^* (arrows in F) compared with *Zic2^+/+^* (asterisks in C). Bleb treatment of *Zic2^Ku/Ku^* embryos yields an F-actin and MHCB distribution in the neuroepithelium (G-I) that closely resembles that in wild-type embryos (A-C), although DLHPs (yellow arrow in A) remain absent from mutants (red arrows in G). (J) Phalloidin intensity profiles (normalised to 100%; from images as in B,E,H) along the basal-to-apical neuroepithelial axis of *Zic2^+/+^* (black) and *Zic2^Ku/Ku^* (pink) uncultured embryos, and *Zic2^Ku/Ku^* embryos exposed to Bleb in culture (blue). Intensity profile scans were performed within a ‘region of interest’ that encompassed at least one-third of each neural hemi-plate. Plot shows the 50% most apical part of each intensity profile, as illustrated by the double arrow in B, between dotted lines marking sub-apical (Sub-Ap) and apical (Ap) positions. (K) Quantitation of areas under the curves (J) for each 10% sector in the 50-100% basal-to-apical neuroepithelial region. Values normalised to wild type (WT) for each 10% sector. *Zic2^Ku/Ku^* embryos show significantly more Phalloidin staining in the 71-80% and 81-90% sectors compared with WT embryos (*P<0.001; repeated measures ANOVA on ranks with post-hoc Tukey tests). This corresponds to the basal extension of F-actin staining in mutants (bracket in E). Bleb-treated embryos do not differ from WT embryos (*P*>0.05), showing rescue of F-actin distribution in mutant neuroepithelium. Scale bars: 30 µm (A,D,G); 12 µm (B,C,E,F,H,I).

*Zic2^Ku/Ku^* embryos with 15-19 somites, soon after spinal neurulation first becomes delayed at the 13- to 14-somite stage, exhibit abnormal F-actin and non-muscle myosin II heavy chain B (MHCB; also known as MYH10) distribution in the spinal neuroepithelium (Fig. 5A-F). Quantitation of staining intensity shows an expansion of the apical Phalloidin-positive domain towards the basal surface (Fig. 5J,K) and a redistribution of MHCB to the apical surface, with intense staining at this location (Fig. S8H). Hence, in terms of abnormal actomyosin accumulation*, Zic2^Ku/Ku^* mutants resemble wild-type embryos subjected to inhibition of Rho kinase (Escuin et al., 2015). In contrast, *Pax3^Sp2H/Sp2H^* embryos, which also fail in spinal closure, have an F-actin and MHCB distribution that is closely similar to that of heterozygous littermate controls (Fig. S8A-F). Hence, the abnormal actomyosin distribution in *Zic2^Ku/Ku^* mutants is not a result of incipient spina bifida, but rather is specific to this NTD model.

### Overactivation of RhoA in *Zic2^Ku/Ku^* mutants

**Fig. 6.**
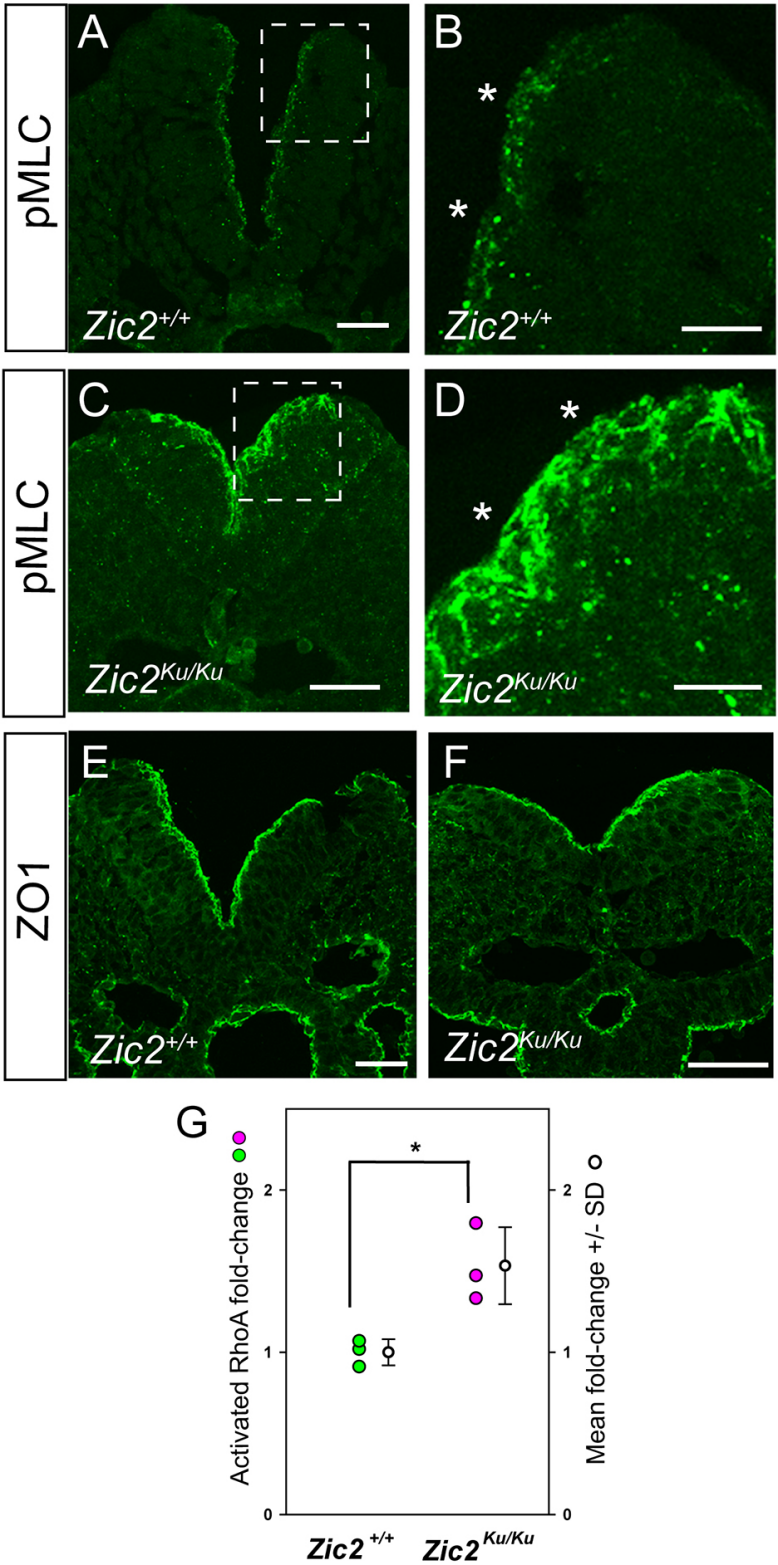
**Overactivation of pMLC and RhoA in the PNP region of *Zic2^Ku/Ku^* embryos.** (A-F) Immunohistochemistry for phospho-myosin light chain (pMLC; A-D) and ZO1 (E,F) in sections through the PNP of *Zic2^+/+^* (A,B,E) and *Zic2^Ku/Ku^* (B,D,F) embryos at 15- to 19-somite stage. Enlargements of boxed areas in A,C show markedly more intense, and less strictly apical, expression of pMLC along the entire dorsoventral extent of the thickened mutant neuroepithelium compared with wild type (asterisks in B,D). In contrast, ZO1 expression is closely similar in apical location and intensity in wild-type and mutant neuroepithelia (E,F). (G) G-LISA assay of active GTP-bound RhoA relative to total RhoA, with mean values normalised to wild type. PNP regions of E9.5 *Zic2^Ku/Ku^* embryos show a significant increase in RhoA activation (*n*=3 biological replicates; *P<0.05; unpaired two-tailed Student's *t*-test;). Scale bars: 30 µm.

To investigate the status of RhoA signalling in the *Zic2^Ku^* mutant, we studied expression of phospho-myosin light chain (pMLC), which is regulated downstream of RhoA (Matsumura, 2005). Immunostaining revealed increased intensity and basal extension of the normally apical pMLC expression in the neuroepithelium of *Zic2^Ku/Ku^* embryos compared with that of wild-type embryos (Fig. 6A-D). In contrast, expression of zonula occludens-1 (ZO1), a marker of epithelial apicobasal polarity (Tornavaca et al., 2015), showed closely similar apical location and intensity in wild-type and mutant neuroepithelia (Fig. 6E,F). The increased pMLC expression suggested an overactivation of RhoA signalling, and this was confirmed by G-LISA assay, with a 1.5-fold increase in RhoA activation in the PNP region of *Zic2^Ku/Ku^* mutants (Fig. 6G). Immunoblotting showed a 6-fold increase in pCofilin in the PNP region of *Zic2^Ku/Ku^* embryos compared with wild-type embryos, but no difference in the remainder of the embryo (data not shown). These findings identify an overactivation of RhoA signalling downstream of the *Zic2^Ku^* mutation. Interestingly, the association with neuroepithelial actomyosin accumulation and failure of spinal neurulation closely resembles that observed after RhoA pathway inhibition (Escuin et al., 2015), suggesting that both overactivation and underactivation of RhoA signalling can be associated with faulty actomyosin distribution in the neuroepithelium, and that RhoA needs to be maintained within strict limits for normal spinal neural tube closure.

### Rescue of closure in *Zic2* mutants by Blebbistatin and Rho kinase inhibition

We found previously, in the context of RhoA signalling inhibition, that accumulation of apical actomyosin and failure of neural tube closure could be prevented by simultaneous exposure of cultured embryos to the myosin inhibitor Blebbistatin (Escuin et al., 2015). To determine whether a similar effect may apply to *Zic2^Ku^*, we cultured mutant embryos in Blebbistatin for 6-8 h from the 12- to 16-somite stage. Mean PNP length in Blebbistatin-treated mutant embryos was significantly reduced compared with that in DMSO-treated control *Zic2^Ku/Ku^* embryos and did not differ significantly from that in wild-type embryos, indicative of normalisation of closure (Fig. 3B). However, the range of PNP lengths extended to larger values in mutants than in wild type, suggesting that rescue was only partial. F-actin and MHCB immunostaining in *Zic2^Ku/Ku^* embryos cultured with Blebbistatin from the 12- to 16-somite stage resembled that in DMSO-treated controls (Fig. 5G-I), with staining intensity analysis confirming the rescue of abnormal actomyosin distribution by Blebbistatin in these embryos (Fig. 5J,K; Fig. S8G,H). If cultures were initiated later, at 17-21 somites, when the PNP was already very large in *Zic2^Ku/Ku^* embryos (Fig. S2), Blebbistatin did not significantly reduce PNP length (Table S2), probably because of pre-existing severe closure delay. Dorsomorphin was also assessed for its effect on actomyosin distribution in cultured *Zic2^Ku/Ku^* embryos, but, in contrast to Blebbistatin, no rescue of F-actin distribution was observed (Fig. S8G).

We found that DLHP formation was not rescued in *Zic2^Ku/Ku^* embryos by Blebbistatin treatment, even though PNP length was diminished at the end of culture (Fig. 3B). The neural folds were relatively straight, without the thickened appearance of untreated mutants (Fig. 4C,D). At an axial level just caudal to the ‘zippering’ point, extremely dorsal bending was detected, just below the neural fold tips (Fig. 4D). We quantified the dorsoventral position of focal bending and found that neuroepithelial bends in Blebbistatin-treated *Zic2^Ku/Ku^* embryos were significantly more dorsal than typical DLHPs in wild-type embryos or in the ‘rescued’ DLHPs of dorsomorphin-treated embryos (Fig. 4F). Hence, Blebbistatin treatment is sufficient to partially rescue the neurulation defects and normalise the actomyosin distribution in *Zic2^Ku/Ku^* embryos, but does not restore typical DLHPs in the neural plate.

**Fig. 7.**
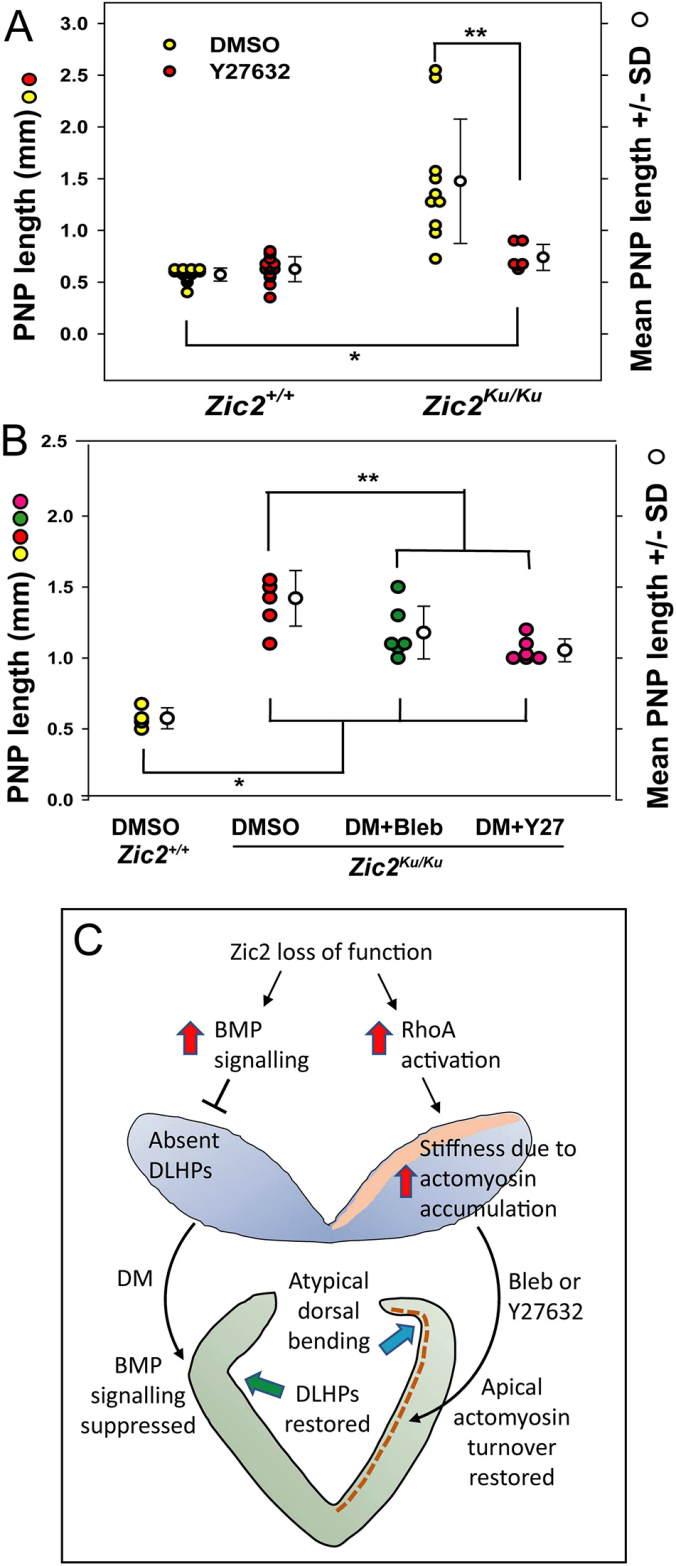
**Partial rescue of neural tube closure in *Zic2^Ku/Ku^* embryos by inhibitor combinations: a dual mechanism of spinal NTDs.** (A) Culture from E8.5 for 18 h in the presence of the Rho kinase inhibitor Y27632 (Y27) leads to significantly reduced PNP length in *Zic2^Ku/Ku^* embryos compared with DMSO-treated mutants (**P<0.003). However, rescue is incomplete and PNP length remains significantly greater in Y27-treated mutants than in DMSO-treated wild-type embryos (*P<0.001). Y27-treated wild-type and mutant embryos do not differ in PNP length (*P*=0.076). (B) DM in combination with Bleb or Y27 similarly reduces PNP length in cultured E8.5 *Zic2^Ku/Ku^* embryos (**P=0.026 for DM+Bleb; **P=0.002 for DM+Y27) but rescue is incomplete in both cases, with treated mutant embryos having significantly greater PNP length than DMSO-treated wild-type embryos (*P<0.001). Two-way ANOVA with Holm-Sidak post-hoc tests. (C) Schematic of the proposed dual mechanism of spinal NTDs in *Zic2^Ku/Ku^* embryos. Loss of Zic2 function releases BMP signalling from its normal regulation by noggin and other endogenous antagonists (left side of diagram). Enhanced BMP signalling suppresses DLHP formation, contributing to failure of PNP closure. This can be ameliorated by DM, an exogenous BMP inhibitor, that reinstates DLHP formation. A second mechanism also operates (right side of diagram), via dysregulated (enhanced) RhoA activation that results in excessive accumulation of actomyosin in the mutant neural plate, leading to stiffening and lack of deformability. Bleb ameliorates the actomyosin accumulation, enhancing closure, although the neural plate shows atypical dorsal bending, not normal DLHPs. A similar effect is seen with Y27632. The mutant neural plate shows dorsoventral thickening, and this appears to be largely rescued by both types of inhibition, although the underlying mechanism is unclear.

In view of the overactivation of RhoA signalling in *Zic2^Ku/Ku^* embryos, we asked whether inhibition of Rho kinase by Y27632 might also rescue the neurulation defects. Indeed, we observed a significant reduction in PNP length in *Zic2^Ku/Ku^* embryos exposed to Y27632, compared with that in DMSO-treated controls (Fig. 7A). Rescue was partial, however, as with dorsomorphin and Blebbistatin, so that Y27632-treated embryos had significantly enlarged PNPs compared with those of DMSO-treated wild type controls.

### Effects of inhibitor combinations in the *Zic2^Ku^* mutant

Although both dorsomorphin and Blebbistatin reduce the delay in PNP closure in *Zic2^Ku/Ku^* embryos, their effects on the neuroepithelium differ markedly: dorsomorphin restored DLHP formation but did not prevent actomyosin accumulation, whereas Blebbistatin failed to restore typical DLHPs but returned the actomyosin distribution to a wild-type appearance. These results suggested that dorsomorphin and Blebbistatin work via different, perhaps complementary, embryonic mechanisms. Therefore, we tested whether a combination of the two types of inhibition could ‘fully’ restore PNP closure in *Zic2^Ku/Ku^* embryos. Exposure to dorsomorphin plus Blebbistatin, or dorsomorphin plus Y27632, both resulted in reduced PNP length after culture, but the extent of this rescue was less than with each inhibitor separately (Fig. 7B). Moreover, dorsomorphin plus Blebbistatin did not restore normal DLHPs, but rather the appearance was closely similar to that of embryos treated with Blebbistatin alone (Fig. 4D,E). This suggests that, rather than being complementary, dorsomorphin and Blebbistatin interfere with each other's action. Blebbistatin, with its effects on the biomechanics of the mutant neuroepithelium appears to have a ‘dominant’ effect over dorsomorphin, with its anti-BMP action. However, each of the inhibitors has an inherent toxicity for cultured embryos, and, although we used sub-toxic concentrations of each, determined through prior dose–response studies, we cannot exclude the possibility that double-inhibitor treatment reduces embryonic viability, thereby contributing to the poorer rescue effect than with single inhibitors.

We conclude that multiple interconnecting signalling pathways are misregulated in *Zic2^Ku/Ku^* mutant embryos (Fig. 7C), each contributing to the final phenotype of arrested spinal neurulation. We can discern distinct functions for BMP signalling in the regulation of DLHP formation, and RhoA signalling in the regulation of actomyosin distribution, both of which exhibit abnormal function in *Zic2^Ku/Ku^* embryos and play a role in opposing the closure of the spinal neural tube.

## DISCUSSION

Here, we investigated the developmental mechanisms underlying failed spinal neural tube closure in the *Zic2^Ku^* mutant, which is one of only a few mouse NTD models that exhibit fully penetrant open spina bifida (Harris and Juriloff, 2010). Our findings reveal a dual molecular mechanism in *Zic2^Ku/Ku^* embryos leading to arrest of PNP closure and spina bifida. BMP signalling, which we previously found to oppose neural plate bending at DLHPs (Ybot-Gonzalez et al., 2007a), is overactivated and DLHPs are absent. Although this defect initially appeared to explain the spinal neurulation phenotype in *Zic2^Ku/Ku^* embryos, we found that use of dorsomorphin to suppress pSmad-1,5,8 expression, and restore DLHP formation, did not provide complete rescue of PNP closure. This indicated the importance of additional mechanisms, and we identified a cytoskeletal defect, involving accumulation of actomyosin apically in the neural plate, downstream of RhoA pathway overactivation in *Zic2^Ku/Ku^* embryos. Suppression of this actomyosin accumulation, using the myosin inhibitor Blebbistatin, provided significant rescue of spinal neural tube closure, despite DLHPs not being restored in the mutant neural plate. Rho kinase inhibition by Y27632 also partially rescued PNP closure in mutants. Hence, two distinct and apparently independent developmental anomalies co-exist in *Zic2^Ku/Ku^* embryos that impede the progression and completion of spinal neurulation.

In order to further unravel the molecular pathogenesis of spina bifida in the *Zic^Ku^* model, it will be important to determine the pathways that link function of Zic2 with regulation of the critical BMP and RhoA signalling pathways that we have identified. Recent years have seen major advances in identification of genes that are regulated by members of the Zic family, based on chromatin immunoprecipitation and other studies (Hatayama and Aruga, 2018). Target DNA sequences include the ‘CTGCTG-core-type’ and the ‘GC-stretch-type’, with CTGCTG-core-type sequences particularly enriched in enhancers throughout the genome. Moreover, Zic2 and other family members can regulate gene expression without DNA binding, through interaction with proteins including the transcription factors Cdx, Oct4 and Pax3, as well as chromatin-remodelling factors (e.g. NuRD and NURF) and nuclear enzymes (e.g. DNA-PK, PARP1 and RNA helicase A).

Developmental signalling pathways regulated by Zic2 include the Shh cascade, via direct interaction with Gli1-3 (Koyabu et al., 2001), the Nodal pathway, via direct binding to Smad2/3 (Houtmeyers et al., 2016), and the Wnt–β-catenin pathway, via interaction with Tcf4 (Pourebrahim et al., 2011). The interaction of Zic2 with Smad proteins, either directly or via indirect interactions [e.g. via binding of Nanog to Gli1 and Smad1 (Li et al., 2016; Suzuki et al., 2006)] could provide a basis for the enhanced BMP signalling that leads to lack of DLHPs in *Zic2^Ku/Ku^* embryos. However, our gene expression findings suggest a primary role for transcriptional downregulation of endogenous BMP inhibitors in mutant embryos. We and others (Warr et al., 2008) find no evidence of a genetic interaction between *Zic2* and *Shh*, despite the possible regulation of Gli1-3 by Zic2. Dysregulation of Wnt–β-catenin signalling could provide another mechanism by which Zic2 loss of function leads to spinal NTDs. Indeed, we previously found that both gain- and loss-of-function β-catenin alleles exacerbate spinal NTDs in mouse embryos heterozygous for loss of *Pax3* function (Palmer et al., 2021). Future studies will be directed towards determining the molecular pathway(s) linking Zic2 and BMP/RhoA signalling during spinal closure.

DLHPs appear towards the end of spinal neurulation (Modes 2 and 3), when the lower levels of the spinal axis are forming. In contrast, early spinal closure (Mode 1) occurs in a neural plate that lacks DLHPs, with bending only at the MHP (Shum and Copp, 1996). DLHPs are precisely located at the mediolateral position along the neural plate where basal contact changes from mesoderm to surface ectoderm, consistent with a biomechanical role in DLHP formation (McShane et al., 2015). Moreover, MHP formation involves adoption of a wedge shaped morphology by all midline neuroepithelial cells, whereas cell wedging is less prominent at the DLHPs (McShane et al., 2015; Schoenwolf and Franks, 1984; Smith et al., 1994).

A recent mechanical finite element modelling study of mammalian spinal neurulation found that DLHPs arise as a largely passive, biomechanical consequence of ‘zippering’ closure (de Goederen et al., 2022). This especially applied at axial levels where the neural plate extends above the mesoderm, as is seen with the thickened *Zic2^Ku/Ku^* neuroepithelium. Hence, this finding would imply that *Zic2^Ku/Ku^* mutants may lack DLHPs as a secondary consequence of zippering failure. Indeed, the cytoskeletal defects we detected in *Zic2^Ku/Ku^* mutants could be the ‘initiator’ of faulty zippering, by reducing the deformability of the neural folds. The lack of zippering would counteract DLHP formation and further enhance the overall neurulation defect. Actomyosin imparts ‘stiffness’ to embryonic tissues (Zhou et al., 2009), and it seems likely that the accumulated actomyosin in the *Zic2* mutant neuroepithelium makes it stiff and resistant to bending and neural fold elevation. Our biomechanical studies provide support for this, by showing that *Zic2^Ku/Ku^* embryos have increased resistance to closure during neurulation (Galea et al., 2017).

Against this interpretation is our finding with Blebbistatin treatment of *Zic2^Ku/Ku^* embryos. Blebbistatin causes enhanced disassembly of F-actin in the *Zic2^Ku/Ku^* neural plate, which likely makes the neural plate more deformable than normal. Indeed, enhanced zippering closure occurred in Blebbistatin-treated embryos, although we did not detect normal DLHP formation, as would be predicted if DLHPs are purely secondary to zippering. Rather, we observed extremely dorsal bending that appeared only shortly before neural fold fusion (i.e. just caudal to the ‘zippering’ point) and which may be a mechanical consequence of the neural folds being ‘pulled’ into apposition by the closed neural tube immediately rostral to this level. Clearly, therefore, the spinal neural tube can undergo zippering closure in the absence of typical DLHPs. We propose, therefore, that BMP signalling strength is a separate regulatory mechanism with regard to DLHP formation (Fig. 7C), so that BMP overactivation directly inhibits DLHPs and prevents their induction by ongoing zippering. Under circumstances in which apical actomyosin accumulation is prevented, as in Blebbistatin-treated *Zic2^Ku/Ku^* embryos, DLHPs cannot be induced because of BMP overactivation, and yet PNP closure can progress, perhaps because the neural folds are made even more deformable than normal, enhancing their closure.

In terms of human genetic disease, *Zic2* loss-of-function mutations are well known to cause the severe brain defect holoprosencephaly (Roessler et al., 2009). This condition can also be caused by Shh mutations (Roessler et al., 1996), and presents as a narrow, univentricular forebrain often associated with close-set eyes or even cyclopia. *Zic2^Ku/Ku^* mouse mutants exhibit holoprosencephaly, but this defect co-exists with failed closure of the cranial neural tube, exencephaly (Elms et al., 2003; Warr et al., 2008). Interestingly, *Shh* mutant mice also exhibit holoprosencephaly, but without exencephaly or spina bifida (Chiang et al., 1996). Although one study reported that human spina bifida can be associated with a polyhistidine tract polymorphism in the *ZIC2* gene (Brown et al., 2002), other studies have failed to find evidence of an association between *ZIC2* polymorphisms and NTDs (Klootwijk et al., 2004; Zhu et al., 2003). It seems likely, therefore, that the value of the *Zic2^Ku^* mutant mouse for an understanding of human NTDs is not as a direct model of primary genetic causation, but rather as a means of understanding how downstream pathways can interact to generate spina bifida, which may also be of importance in human defects. Several decades of genetic research on human NTDs have shown that these conditions are rarely single-gene disorders, but result from oligogenic inheritance together with important environmental influences (Chen et al., 2018; Greene et al., 2009). Our findings suggest that genetic variants that lead to enhanced BMP and RhoA signalling could be candidates for causation of human NTDs, as in the *Zic2^Ku^* mutant mouse.

## MATERIALS AND METHODS

### Breeding and genotyping of mouse strains

Mouse studies were conducted under auspices of the UK Animals (Scientific Procedures) Act 1986 and the National Centre for the 3Rs' Responsibility in the Use of Animals for Medical Research (2019). Mutant strains were *Zic2-Kumba* (*Zic2^Ku^*), *splotch* (*Pax3^Sp2H^*) and Sonic hedgehog-null (*Shh^tm1Chg^*), all maintained as closed random-bred colonies, apart from *Zic2^Ku^*, in which heterozygotes on the C57BL/6 background were outcrossed to C3H/He for a single generation, after which *Zic2^Ku/+^* F1 mice were intercrossed to generate F2 embryos for experiments. Experimental litters containing *Pax3^Sp2H/Sp2H^* embryos were generated by heterozygote matings. Doubly mutant *Zic2^Ku/+^*; *Shh^+/−^* mice were bred from matings between the two strains, and then intercrossed to yield litters for the *Zic2-Shh* interaction study. Strains were genotyped as described (Burren et al., 2008; Chiang et al., 1996; Ybot-Gonzalez et al., 2007a) using ear-punch DNA from pre-weaning pups and yolk sac DNA from embryos. Non-mutant embryos from random-bred CD1 mice were used for *in situ* hybridisation studies of *Zic2* gene expression (Fig. S3).

### Embryo collection, culture and processing

Pregnant females were killed at E8.5-E9.5 (E0.5 is noon on the day of finding a copulation plug after overnight mating). Embryos were dissected in Dulbecco's modified Eagle medium (Invitrogen) containing 10% fetal bovine serum (Sigma-Aldrich). Following whole-embryo culture (Copp et al., 2000), or immediately after dissection of non-cultured embryos, somites were counted and PNP length was measured by eyepiece graticule on a Zeiss SV11 stereomicroscope. PNP and ‘body’ fragments were generated by cutting rostral to the most recently formed somite pair. Embryos were fixed in 4% paraformaldehyde (PFA) in phosphate buffered saline (PBS) for 1-4 h, or overnight in Bouin's fluid (for histology) and then processed for: (1) cryosectioning, by embedding in 7.5% gelatine in 15% sucrose/PBS; (2) paraffin sectioning, by dehydration through ethanols and Histo-Clear following by embedding in 56°C paraffin wax; (3) whole-mount *in situ* hybridisation, by washes in diethylpyrocarbonate (DEPC)-treated PBS, followed by dehydration through a methanol series and storage in 100% methanol at −20°C; (4) protein extraction, by rinsing twice in ice-cold PBS, followed by snap freezing on liquid N_2_ and storage at −80°C.

### Chemical inhibitors

Inhibitors were prepared as stock solutions in DMSO and stored as frozen aliquots: dorsomorphin (04-0024, Reprocell; 10 mM stock), Blebbistatin (203390, Calbiochem; 250 mM) and Y27632 (688000, Calbiochem; 5 mM). Immediately prior to use, each stock was diluted with DMSO and added to embryo culture serum to a maximum of 0.1% (v/v). Final concentrations were as follows: dorsomorphin, 5 µM; Blebbistatin, 50 µM; Y27632, 5 µM. An equal volume of DMSO was added to control cultures. Cultures were performed in the dark to avoid photo-inactivation of inhibitors.

### RhoA activation assay and western blotting

Activated RhoA was determined using a RhoA G-LISA Activation Assay kit (BK124, Cytoskeleton Inc.) according to the manufacturer's instructions (Escuin et al., 2015). Total RhoA was determined by enzyme-linked immunosorbent assay (ELISA; BK150, Cytoskeleton Inc.). Assays were performed in duplicate on three biological replicates for each genotype (wild-type and mutant). Protein extraction, western blotting, antibody detection, autoradiography and densitometry were performed as described previously (Escuin et al., 2015). Anti-Cofilin (sc-8441, Santa Cruz Biotechnology; 1:1000) and anti-pCofilin (3313, Cell Signaling Technology; 1:1000) were used for immunodetection.

### Whole-mount situ hybridisation

Riboprobes for *in situ* hybridisation were as described: *Bmp2* and *Bmp4* (Furuta et al., 1997), *Bmp7* (Arkell and Beddington, 1997), *Cdh6* (Henderson et al., 1997), chordin (Klingensmith et al., 1999), *Gli1-3* (Hui and Joyner, 1993), *Msx1* (Mitsiadis et al., 2003), neuralin (Coffinier et al., 2001), noggin (Ybot-Gonzalez et al., 2007a), *Ptc* (Goodrich et al., 1997), *Shh* (Echelard et al., 1993) and *Zic2* (Gaston-Massuet et al., 2005).

Embryos were rehydrated from methanol, washed 2× in PBS containing 0.1% (v/v) Tween-20 (PBT), bleached in 6% (v/v) hydrogen peroxide in PBT on ice for 1 h, washed 3× in PBT, permeabilised with proteinase K (5 μg/ml) at room temperature (RT) for 1 min (E8.5) or 2 min (E9.5), then washed in glycine (2 mg/ml) in PBT, with two further washes in PBT. Embryos were re-fixed in 0.2% (v/v) glutaraldehyde in 4% (w/v) PFA in DEPC-PBS for 20 min at RT, washed twice in PBT, then pre-hybridised with gentle rocking for at least 3 h in 50% formamide, 5× SSC (pH 4.5), 1% sodium dodecyl sulphate, 50 μg/ml yeast RNA, 50 μg/ml heparin at 65-70°C. Hybridisation was overnight at 70°C with gentle rocking, in pre-hybridisation solution plus digoxigenin-labelled RNA probe, 1 μg/ml. Embryos were washed three times in solution 1 [50% formamide, 5× SSC (pH 4.5), 1% SDS in DEPC-water] at 70°C for 15-30 min, twice in solution 2 [50% formamide, 2× SSC (pH 4.5), 1% SDS in DEPC-water] at 65°C for 30 min each, then three times in Tris-buffered saline containing Tween 20 [TBSTw; 140 mM NaCl, 2.7 mM KCl, 25 mM Tris-HCl (pH 7.5), 2 mM tetramisole hydrochloride, 1% (v/v) Tween 20]. Incubation with shaking in 10% heat-inactivated sheep serum in TBSTw for 90 min at RT was followed by overnight incubation at 4°C in sheep anti-digoxigenin Fab fragments conjugated with alkaline phosphatase (Roche), diluted 1:2000 in TBSTw with 1% heat-inactivated sheep serum. After overnight washes with shaking in TBSTw, embryos were washed for 3×10 min at RT in NTMT solution [100 mM NaCl, 100 mM Tris HCl (pH 9.5), 50 mM MgCl_2_, 2 mM tetramisole hydrochloride, 1% Tween 20 in water] and then NTMT solution containing 4.5 μl/ml nitro blue tetrazolium (NBT) and 3.5 μl/ml 5-bromo-4-chloro-3-indolyl phosphate (BCIP) at RT with shaking. Developing was for 5-18 h depending on the probe, with control embryos developed for the same time. Embryos were washed three times in PBT, re-fixed at RT in 4% PFA at 4°C and stored protected from light at 4°C in PBT plus thimerosal. Hybridised embryos were embedded in gelatine/albumin and vibratome sectioned at 50 μm (Copp et al., 2000).

### Immunohistochemistry on cryosections

Gelatine was removed from cryosections by incubation in PBS for 30 min at 37°C. Samples were blocked and permeabilised in 10% sheep serum, 0.1% Tween 20 in PBS, incubated with primary antibody overnight at 4°C, rinsed in PBS, and incubated with secondary antibody for 1 h at RT. For F-actin, sections were incubated in Phalloidin (Alexa Fluor 568-Phalloidin, A12380, Life Technologies) for 1 h at RT. Samples were washed with DAPI (4′,6-diamidino-2-phenylindole) and mounted in Mowiol 4-88 mounting medium (Sigma-Aldrich; prepared with glycerol and 0.2 M Tris-HCl pH 6.8). Primary antibodies were anti-pSmad-1,5,8 (9511, Cell Signaling Technology; 1:50), anti-MHCB (PRB-445P, Covance; 1:500), anti-pMLC (3671, Cell Signaling Technology; 1:50) and anti-ZO1 (40-2200, Life Technologies; 1:50). Secondary antibody was Alexa Fluor 488 goat anti-rabbit IgG (A11070, Life Technologies; 1:500). Labelled cells were examined and quantified by epifluorescence on an inverted LSM710 confocal system mounted on an Axio Observer Z1 microscope (Carl Zeiss, UK). Images were acquired at RT using a 63× oil immersion objective. The thickness of optical sections was set at 0.2-0.9 µm. The Alexa Fluor 488 dye was excited by a 488 nm line of an Argon laser and Alexa Fluor 568 by a 561 nm diode laser. *Z*-projections of confocal stacks were created in ImageJ. Images were further processed in Photoshop CS3 (Adobe).

### Immunohistochemistry on paraffin sections

Slides were incubated in Histo-Clear, then rehydrated through an ethanol series. Antigens were unmasked by boiling slides in 50 mM sodium citrate tribasic, pH 6.0. Following three washes in Tris-buffered saline (pH 7.4) containing 0.2% Triton X-100 (TBST), slides were blocked at RT for 90 min in 2 mg/ml bovine serum albumin (BSA; Cohn V fraction), 0.15% glycine, 10% heat-inactivated sheep serum in TBST. Overnight incubation of slides at 4°C in primary antibody diluted in 2 mg/ml BSA, 0.15% glycine and 5% heat-inactivated sheep serum in TBST was followed by three TBST washes, then incubation for 1 h at RT in secondary antibody diluted as for primary. Slides were washed in TBST, then incubated in DAPI diluted 1:10,000 in TBST for 5 min at RT. After two further TBST washes, sections were mounted in 30% (w/v) glycerol, 12% (w/v) Mowiol 4-88 and 2.5% 1,4-diazobicyclo(2.2.2)octane (DABCO) in 10 mM Tris-HCl (pH 6.8) and stored at 4°C.

Primary antibodies were anti-Isl1 [40.2D6, Developmental Studies Hybridoma Bank at the University of Iowa (DSHB); 1:50], anti-Nkx6.1 (F55A10, DSHB; 1:50) and anti-Pax3 (DSHB; 1:50). Secondary antibodies were Alexa Fluor 568 goat (A21069, Invitrogen; 1:400) or donkey (A10037, Invitrogen; 1:400) anti-mouse IgG, and Alexa Fluor 488 goat (A-11070, Invitrogen; 1:400) or donkey (A-21202, Invitrogen; 1:400) anti-mouse IgG.

### Image analysis and quantification of Phalloidin and MHCB staining

Between three and five adjacent Phalloidin- and anti-MHCB-stained sections were analysed per embryo. For each section and each side of the neural plate, a region of interest (ROI) was outlined, comprising the full thickness of the neural plate along ∼30% of the dorsoventral extent of one hemi-plate. The fluorescence intensity within the ROI was measured using the Image function ‘Plot Profile’ and integrated into a custom-written ImageJ macro, as described (Escuin et al., 2015).

### Statistical analysis

All graph production and statistical analysis were performed using Sigmaplot v 14.5 and SigmaStat v1.1 (Systat Software Inc). At least three independent experiments were performed for each assay.

## Supplementary Material

10.1242/dmm.049858_sup1 Supplementary information
