# Supplementary material for: Dual mechanism underlying failure of neural tube closure in the Zic2 mutant mouse
Source: Dis Model Mech. 2023 Mar 14;16(3):dmm049858. doi: 10.1242/dmm.049858 (PMC10073009; doi:10.1242/dmm.049858)
Supplement: Supplementary information [file dmm-16-049858-s1.pdf]

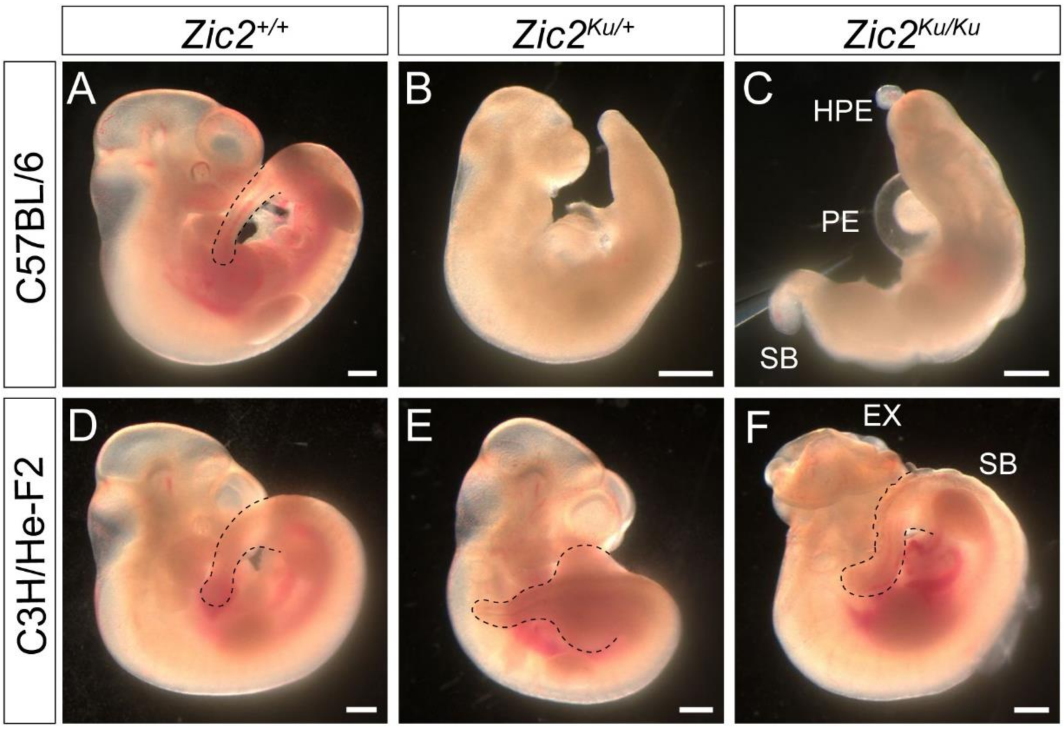

Fig. S1. Variation in severity of *Zic2*$^{Ku/Ku}$ phenotypes on C57BL/6 genetic background and after outcross to C3H/He. Embryos at E10.5 showing typical phenotypes of *Zic2*$^{+/+}$ (A,D), *Zic2*$^{Ku/+}$ (B,E) and *Zic2*$^{Ku/Ku}$ (C,F) genotypes, on the original C57BL/6 congenic background (A-C) and after a single outcross to C3H/He followed by an intercross of heterozygotes (D-F; C3H/He-F2). Wild-type embryos appear normal (A,D), while heterozygotes are normal on the C3H/He-F2 background (E), but can be growth retarded on C57BL/6 (B). Among homozygotes, open spina bifida is present in 100% of cases on both backgrounds, with exencephaly (EX: C57BL/6, 67%; C3H/He-F2, 74%; see F) or holoprosencephaly (HPE: C57BL/6, 13%; C3H/He-F2, 22%; see C) as the main cranial phenotypes. Severity of growth retardation among homozygotes is greater on C57BL/6 than C3H/He-F2. Most *Zic2*$^{Ku/Ku}$ embryos die by E11.5 on C57BL/6 with frequent pericardial oedema (PE in C), whereas *Zic2*$^{Ku/Ku}$ embryos on C3H/He-F2 appear healthier (F) and typically survive to E13.5. Mean litter size ($\pm$ SEM): 6.3 $\pm$ 0.3 on C57BL/6 and 8.2 $\pm$ 0.2 on C3H/He-F2, with 58% of mated females (n = 99) proving non-pregnant on C57BL/6 compared with 16% (n = 105) on C3H/He-F2. For these reasons, analysis of the mechanisms underlying the *Zic2*$^{Ku/Ku}$ phenotype was not possible on the C57BL/6 congenic background, and instead embryos on the C3H/He-F2 background were used throughout the study. Scale bars: 0.5 mm.

Disease Models & Mechanisms • Supplementary information

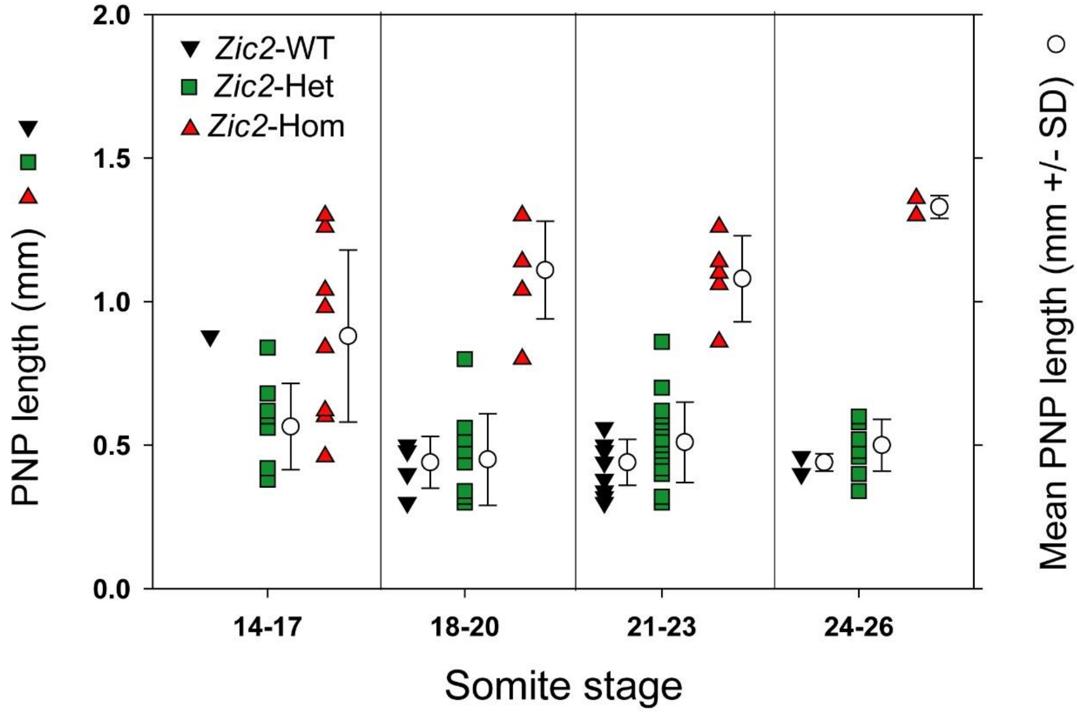

Fig. S2. Progressive delay of PNP closure in *Zic2*$^{Ku/Ku}$ embryos. Litters from matings between heterozygotes were explanted at E8.5 and cultured for 16 h. PNP length was measured blind to genotype, which was determined subsequently. Embryos at the start of culture (n = 97) were of varying stage, reflecting inter- and intra-litter variability. With an average increase of 8 somites in the culture period, embryos had ~14-24 somites when analysed, and were grouped into four somite-ranges. PNP length overlaps between the three genotypes at 14-17 somites, whereas there is an almost 3-fold greater PNP length in homozygotes compared with wild-type and heterozygotes at 24-26 somites. Two-way ANOVA shows significant variation between genotypes ($p < 0.001$) and significant interaction with somite stage ($p < 0.001$). Post-hoc Holm-Sidak tests reveal significantly greater PNP length in homozygotes at 21-23 and 24-26 somites, compared with 14-17 somites ($p < 0.05$). Homozygotes have significantly greater PNP lengths than wild-type and heterozygous embryos at 18-20, 21-23 and 24-26 somite stages ($p < 0.05$).

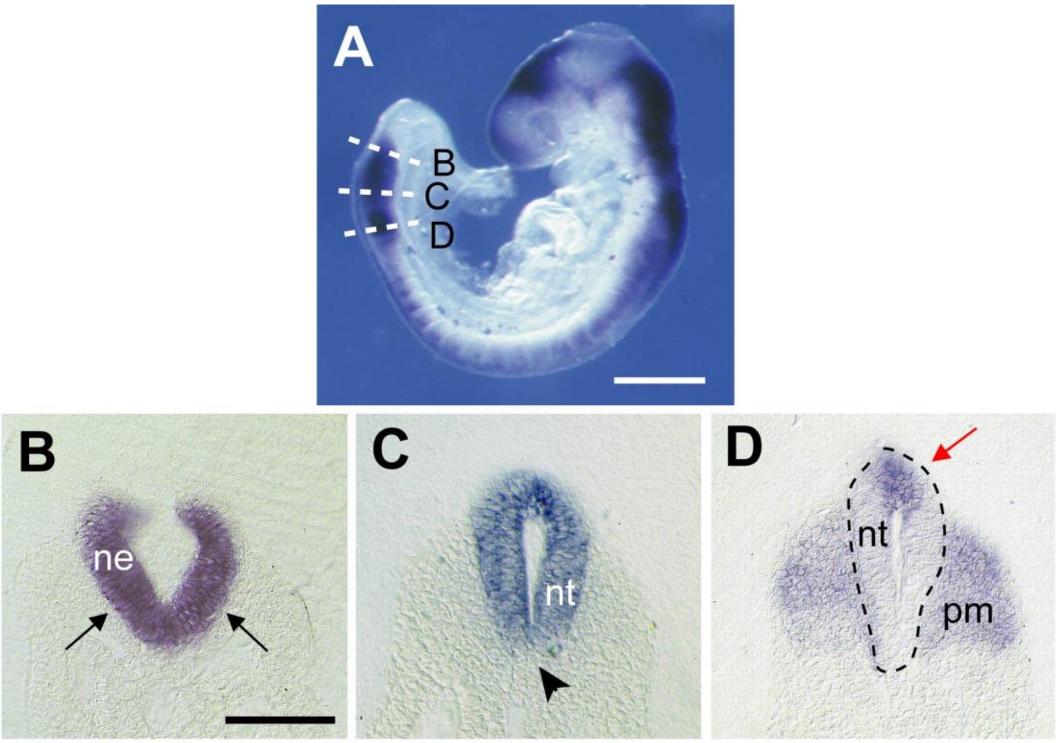

Fig. S3. Expression of *Zic2* during and following normal spinal neural tube closure. Whole mount in situ hybridisation (A) and vibratome transverse sections (B-D) through the caudal region of a non-mutant CD1 embryo (n = 4) at E9.5, with section planes in B-D as indicated by dashed lines in A. During PNP closure (B), *Zic2* transcripts are restricted to the neuroepithelium (ne) which is entirely positive (arrows in B). Immediately following closure (C), transcripts continue to be restricted to the neural tube (nt) with most cells expressing *Zic2*, although floor plate cells appear negative (arrowhead in C). More rostrally, just caudal to the somite rows (D), *Zic2* shows dorsalised neural tube expression, with transcripts now confined to the future roof plate (red arrow in D). Dashed lines outline the neural tube. The rostral pre-somitic mesoderm (pm), which contains incipient somites, is positive for *Zic2* at this level. Scale bars: 0.5 mm in A; 0.2 mm in B (also C, D).

Disease Models & Mechanisms • Supplementary information

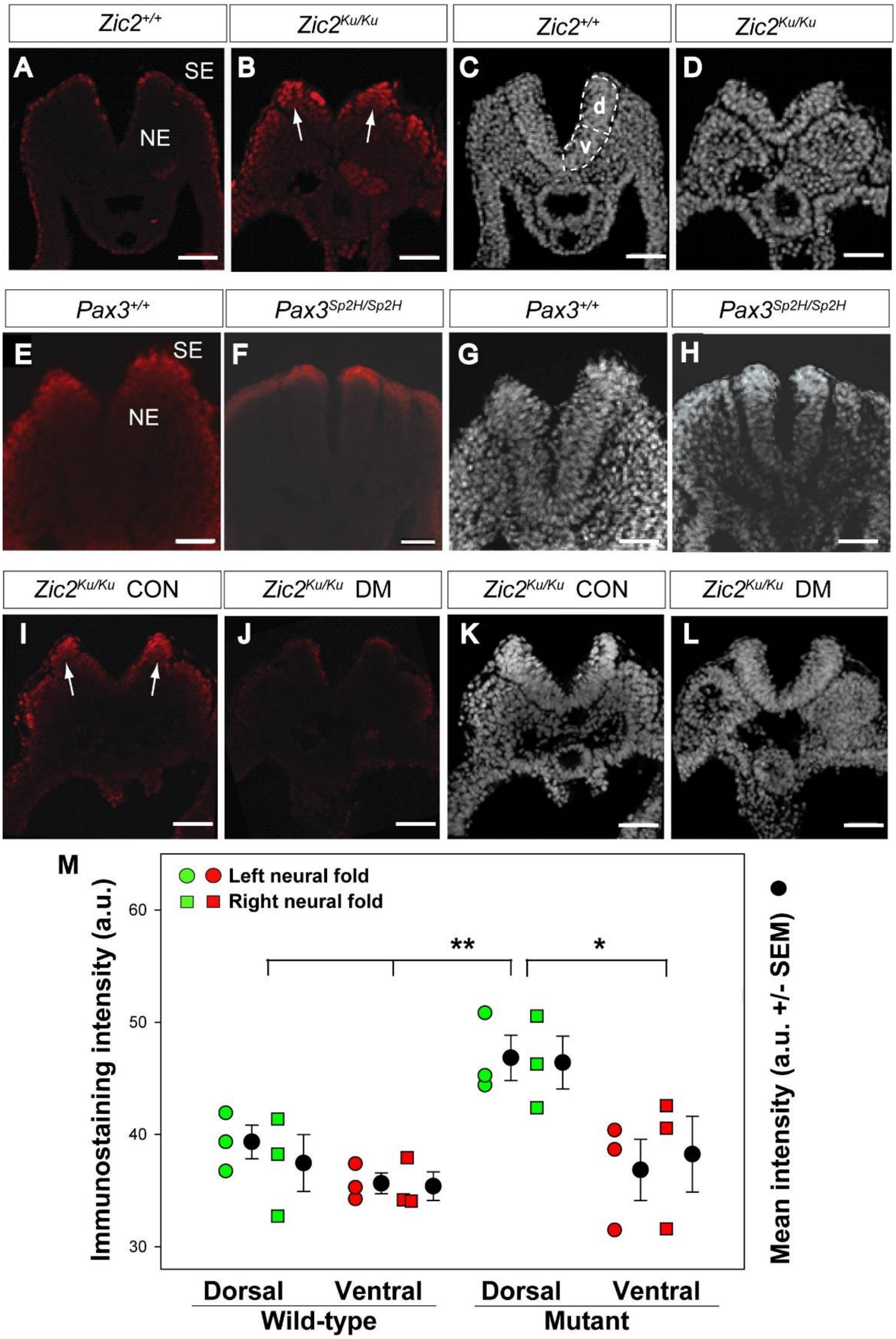

Fig. S4. Expression of pSmad-1,5,8 in *Zic2*^{Ku} and *Pax3*^{Sp2H} embryos.
Immunohistochemistry for pSmad-1,5,8 (A,B,E,F,I,J) and corresponding DAPI images (C,D,G,H,K,L) in *Zic2*$^{+/+}$ (A,C), *Zic2*$^{Ku/Ku}$ (B,D,I-L), *Pax3*$^{+/+}$ (E,G) and *Pax3*$^{Sp2H/Sp2H}$ (F,H)

Disease Models & Mechanisms • Supplementary information

embryos at E9.5. (**A-D**) Additional embryos to those shown in Fig 1H,I, demonstrating enhanced pSmad-1,5,8 expression in the neuroepithelium (NE) and dorsal surface ectoderm (SE) of *Zic2*$^{Ku/Ku}$ (arrows in B) compared with wild-type (A). (**E-H**) *Pax3*$^{+/+}$ and *Pax3*$^{Sp2H/Sp2H}$ E9.5 embryos show similar pSmad-1,5,8 expression, confined mainly to the SE. Like *Zic2*$^{Ku/Ku}$ embryos, spinal (PNP) neural tube closure fails in 100% of *Pax3*$^{Sp2H/Sp2H}$ embryos. Hence, enhanced pSmad-1,5,8 expression is not a result of incipient closure failure *per se*. (**I-L**) Additional embryos to those in Fig 1J,K, showing reduced pSmad-1,5,8 expression in *Zic2*$^{Ku/Ku}$ embryo exposed for 16 h from E8.5 in embryo culture to 5 µM dorsomorphin (DM)(J) compared with vehicle-treated control *Zic2*$^{Ku/Ku}$ embryo (CON)(I). (**M**) Smad-1,5,8 immunostaining intensity comparison between equally sized dorsal (d) and ventral (v) regions of each neural hemi-plates (see outlined areas in C) of *Zic2*$^{+/+}$ and *Zic2*$^{Ku/Ku}$ non-cultured embryos. Left and right neural folds were analysed in a single section of 3 different embryos of each genotype. Three-way ANOVA: Smad-1,5,8 expression is significantly greater in mutant than wild-type (** $p = 0.005$), and in dorsal than ventral regions (* $p = 0.002$) whereas left and right neural folds do not differ ($p > 0.05$). Parallel analysis of DAPI staining in the same sections shows no significant differences. Scale bars: 50 µm.

Disease Models & Mechanisms • Supplementary information

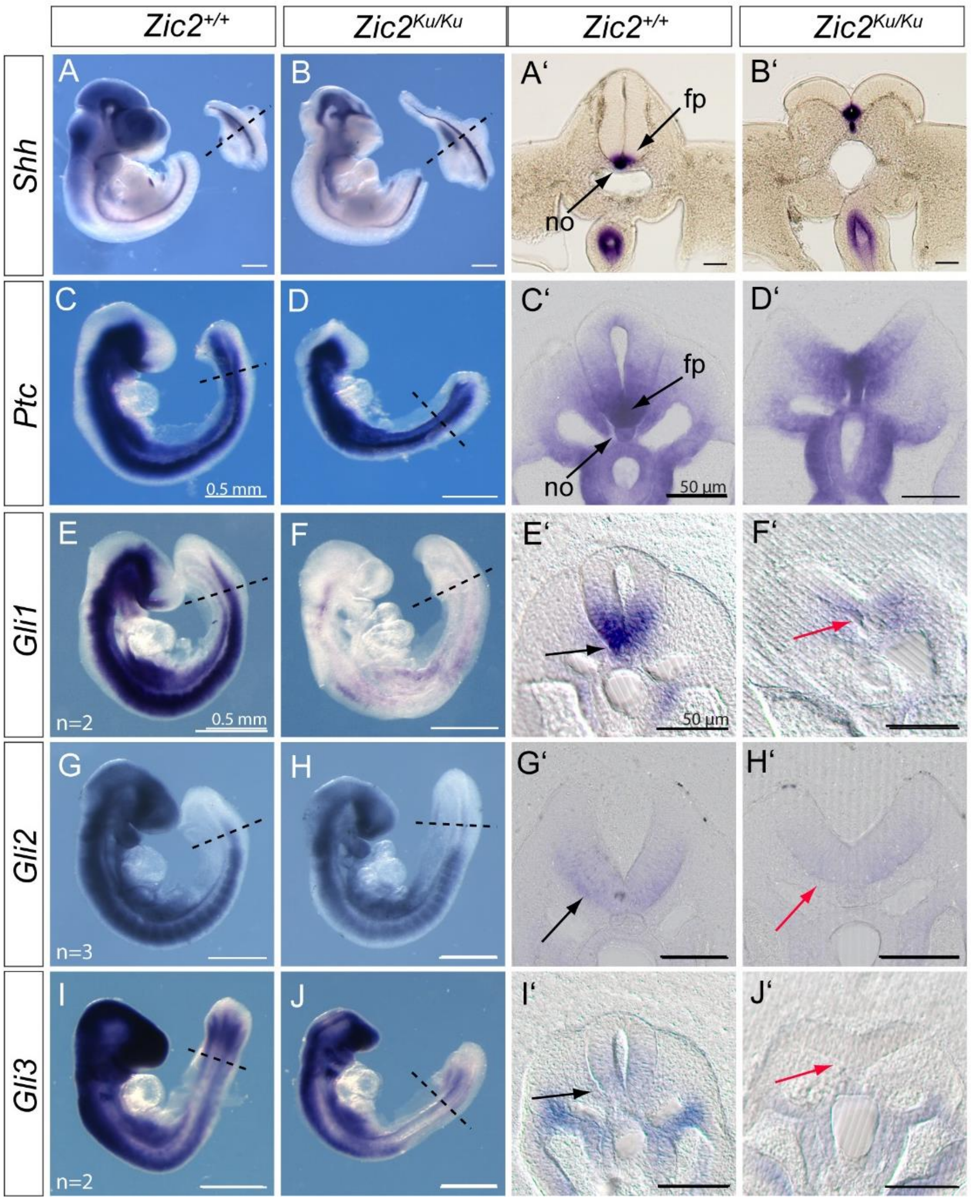

Fig. S5. Expression of Shh pathway genes in *Zic2*$^{+/+}$ and *Zic2*$^{Ku/Ku}$ embryos. Whole mount in situ hybridisation for *Shh* (A,B), *Ptc* (C,D), *Gli1* (E,F), *Gli2* (G,H) and *Gli3* (I,J), with vibratome sections through the closed (A',C',E',G',I') or persistently open spinal neural tube (B',D',F',H',J'). *Zic2*$^{+/+}$ (A,C,E,G,I) and *Zic2*$^{Ku/Ku}$ (B,D,F,H,J) embryos are shown at E10.5 (A,B) and E9.5 (C-J). Dashed lines: planes of section as in right hand panels. Expression of *Shh* and *Ptc* in neural tube floor plate (fp) and notochord (no) are closely similar in *Zic2*$^{+/+}$ and *Zic2*$^{Ku/Ku}$ (A-D), despite the open neural tube in mutants (B',D'). *Gli1* expression is markedly down-regulated in *Zic2*$^{Ku/Ku}$ embryos (F) compared with *Zic2*$^{+/+}$ (E,F; see black and red arrows in E',F'). In contrast, *Gli2* and *Gli3* expression is more similar between genotypes (G-J; black and red arrows in G'-J'). Three or more embryos were hybridized per genotype and probe combination, except where indicated. Scale bars: 0.5 mm, A-J; 50 µm, A'-J'.

Disease Models & Mechanisms • Supplementary information

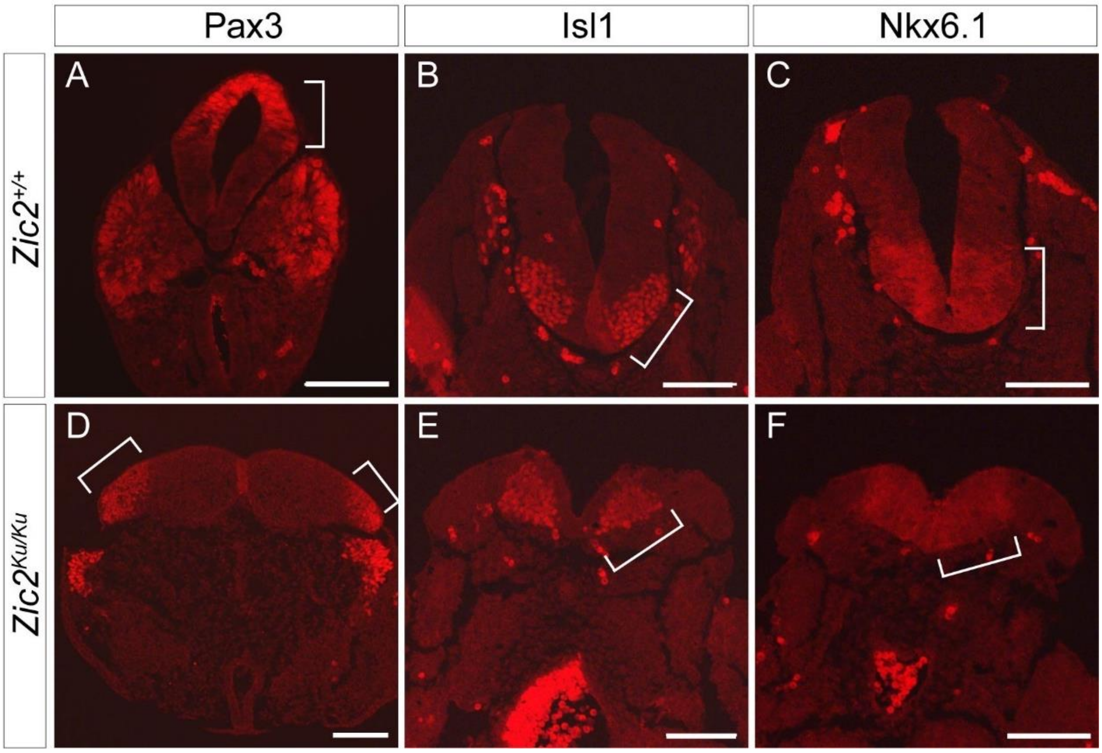

Fig. S6. Normal dorsoventral patterning of the neural tube in *Zic2$^{Ku/Ku}$* embryos. Immuno-histochemistry for dorsoventral markers on transverse sections of E10.5 Zic2$^{+/+}$ (A-C) and Zic2$^{Ku/Ku}$ (D-F) embryos. Despite the open neural tube of mutant embryos, the dorsoventral patterning of protein expression (indicated by brackets) is maintained, with Pax3 in the dorsal neuroepithelium and dermatomyotome (A,D), Isl1 in the ventrolateral motor neuron domain (B,D) and Nkx6.1 in the ventral neuroepithelium (C,E). Scale bars: 100 µm.

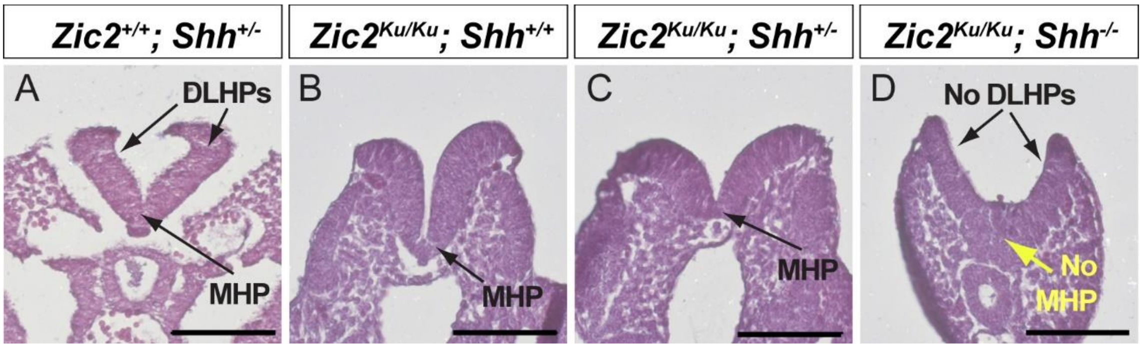

Fig. S7. Deletion of *Shh* on the *Zic2*$^{Ku/Ku}$ background does not restore DLHPs. Transverse paraffin sections, stained with haematoxylin and eosin, through the PNP of E9.5-E10.5 double mutants with various combinations of *Zic2*^{Ku} and *Shh*-null alleles. (**A,B**) DLHPs are present in the PNP of a control *Zic2*$^{+/+}$*; Shh*$^{+/-}$ embryo, but absent from *Zic2*$^{Ku/Ku}$*; Shh*$^{+/+}$ embryos (B). (**C**) Removal of one allele of *Shh* from *Zic2*$^{Ku/Ku}$ embryos fails to restore DLHP formation, and spina bifida occurred in all embryos (n = 16) of this genotype (see Table S1). (**D**) Removal of both alleles of *Shh* from *Zic2*$^{Ku/Ku}$ embryos produces a somewhat different morphological appearance in which there is no distinct median hinge point (MHP, yellow arrow in D). This is secondary to absence of the notochord in Shh loss-of-function, and closely resembles the PNP morphology in *Shh*$^{-/-}$ embryos, as described previously (Ybot-Gonzalez et al. 2002. Development 129, 2507-2517). The neural folds are not splayed apart as in *Zic2*$^{Ku/Ku}$ embryos with persisting Shh function (B,C), but the *Zic2*$^{Ku/Ku}$*; Shh*$^{-/-}$ genotype lacks DLHPs (black arrows in D), reflecting lack of DLHP rescue by removal of Shh function. A single E10.5 embryo of this genotype was obtained and this had spina bifida (Table S1), consistent with lack of DLHP rescue. n = 2-4 embryos sectioned per genotype. Scale bars: 50 μm.

Disease Models & Mechanisms • Supplementary information

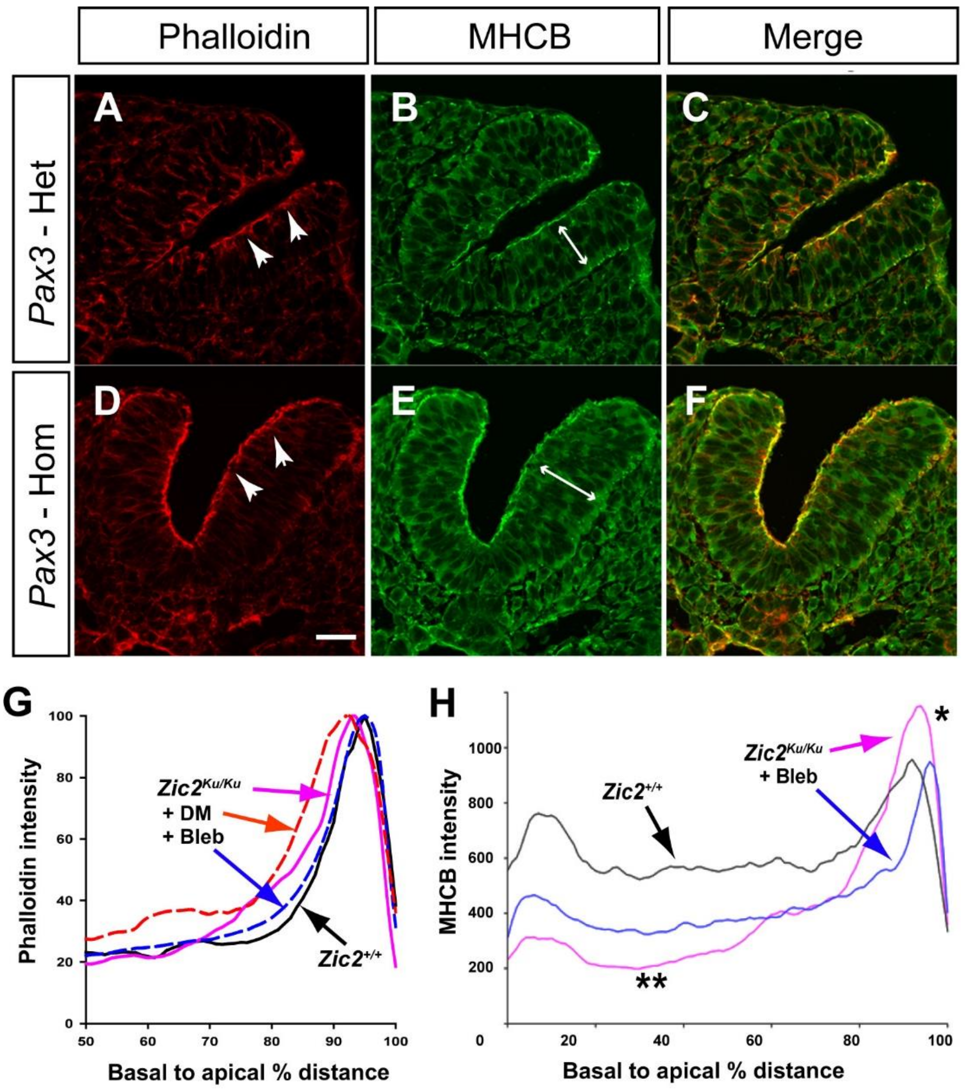

Fig. S8. Distribution of F-actin and myosin heavy chain B (MHCB) do not differ between neuroepithelia of Pax3 heterozygous and mutant embryos. (**A-F**) Transverse sections through the posterior neuropore of $Pax3^{Sp2H/+}$ heterozygous (A-C) and $Pax3^{Sp2H/Sp2H}$ homozygous (D-F) embryos at E9.5, stained with phalloidin (A,D; red) or anti-MHCB (B,E; green). C,F: merged images. Pax3 heterozygotes complete neural tube closure in > 95% of cases, whereas homozygotes exhibit 100% spina bifida. Despite this different developmental outcome, the apical localization of F-actin (arrowheads in A,D) and apico-basal distribution of MHCB (double-headed arrows in B,E) are closely similar between genotypes. Scale bar: 30 µm. (**G**) Phalloidin intensity profiles (normalised) along the 50% apical-most part of the basal-to-apical neuroepithelial axis of $Zic2^{+/+}$ (black) and $Zic2^{Ku/Ku}$ (pink) embryos. Data are the same as in Fig 5J, with the addition of $Zic2^{Ku/Ku}$ embryos exposed to dorsomorphin (DM) in culture (orange). Whereas Bleb treatment (blue) yields a mutant F-actin profile similar to wild-type, DM does not rescue the mutant profile. (**H**) MHCB intensity profile (non-

normalised) along the full basal-to-apical axis of the neuroepithelium, obtained from images as in Fig 5C,F,I. Note the enhanced apical-most signal (*) in *Zic2*$^{Ku/Ku}$ embryos (pink) which is abolished in mutants treated with Bleb in culture (blue) so they resemble *Zic2*$^{+/+}$ (black). Diminished basal MHCB (**) is also seen in untreated mutants and this is partially rescued by Bleb.

Disease Models & Mechanisms • Supplementary information

Table S1. Reduction in Shh function does not rescue spina bifida in *Zic2*$^{Ku/Ku}$ mutants

Zic2 genotype	Shh genotype	Expected % genotype frequency	Observed % genotype frequency * (no. embryos **)	Spina bifida % frequency (no. SB/total embryos ***)
+/+	+/+	6.25	3.0 (2)	0 (0/0)
+/+	+/-	12.5	14.9 (10)	0 (0/6)
+/+	-/-	6.25	3.0 (2)	0 (0/2)
Ku/+	+/+	12.5	1.5 (1)	0 (0/1)
Ku/+	+/-	25.0	41.8 (28)	26.3 (5/19)
Ku/+	-/-	12.5	3.0 (2)	0 (0/2)
Ku/Ku	+/+	6.25	3.0 (2)	100 (1/1)
Ku/Ku	+/-	12.5	25.4 (17)	100 (16/16)
Ku/Ku	-/-	6.25	4.5 (3)	100 (1/1)

* Observed genotype frequencies do not differ from expected (chi-square test; $p > 0.05$).
** Genotype frequencies are based on embryos collected at E9.5 and E10.5, combined (n = 67).
*** Spina bifida (SB) frequencies are based solely on embryos collected at E10.5 (n = 48), when spinal closure has been completed in normal development.

Disease Models & Mechanisms • Supplementary information

Table S2. Culture of *Zic2* wild-type and mutant embryos for 6-8 h from the 17-21 somite stage in the presence of Blebbistatin or DMSO as control

Genotype	Treatment	PNP length (mean ± SD)	no. embryos	p-value
Zic2$^{+/+}$	DMSO	0.45 ± 0.15	6	0.573 *
	Blebbistatin	0.49 ± 0.10	6	
Zic2$^{Ku/Ku}$	DMSO	1.81 ± 0.14	4	0.670 *
	Blebbistatin	1.77 ± 0.18	5	

* Effect of Blebbistatin is non-significant (t-tests) in both wild-type and mutant genotypes.
